# Investigation of the putative mechanism by which *Ferula sinkiangensis* leaves alleviate functional Dyspepsia–like symptoms in rats via modulation of the NO/cGMP/PKG signaling pathway

**DOI:** 10.3389/fphar.2026.1843817

**Published:** 2026-09-01

**Authors:** Xuliang Xia, Dexi Wang, Jing Liu, Yu Liu, Haiying Zhang, Buerlan Jianaer, Fangfang Fan, Shengjun Zhao

**Affiliations:** 1 Graduate School, the Fourth Clinical College of Xinjiang Medical University, Urumqi, China; 2 Department of Pharmacy, Affiliated Hospital of Traditional Chinese Medicine of Xinjiang Medical University, Urumqi, China; 3 Xinjiang Key Laboratory of Processing and Research of Traditional Chinese Medicine, Urumqi, China; 4 Office of Drug Clinical Trial Institution, Affiliated Hospital of Traditional Chinese Medicine of Xinjiang Medical University, Urumqi, China

**Keywords:** Ferula sinkiangensis, functional dyspepsia, gastric emptying, network pharmacology, NO/cGMP/PKG pathway

## Abstract

Functional dyspepsia (FD) is a common functional gastrointestinal disorder with limited therapeutic options that often cause adverse effects. *Ferula sinkiangensis* (*F. sinkiangensis*) leaves have been used traditionally in Xinjiang, China, to relieve gastrointestinal complaints, yet their pharmacodynamic efficacy, active constituents, and molecular mechanisms against FD remain poorly defined. In a prior study, we detected 23 systemically absorbed prototype components of *F. sinkiangensis* leaf aqueous extract (FSLAE) in rats by ultra-high-performance liquid chromatography coupled with quadrupole time-of-flight mass spectrometry (UPLC-Q-TOF-MS). Building on those results, the present study assessed FSLAE’s potential therapeutic effects against FD and investigated its molecular mechanisms based on the previously characterized components. We applied network pharmacology to identify putative core active ingredients, hub targets, and relevant signaling pathways, and then used molecular docking to predict binding interactions between core ingredients and key targets. Finally, we evaluated FSLAE’s pharmacodynamic effects and mechanisms in a rat model of FD induced by iodoacetamide plus chronic tail-clamping stress. Network pharmacology highlighted the NO/cGMP/PKG pathway as a likely principal regulatory route, and five core ingredients showed favorable predicted binding affinities for eNOS and PKG1. FSLAE increased gastrointestinal motility in a dose-dependent manner, reduced gastric mucosal injury, restored gastrointestinal hormone balance, and lowered inflammatory cytokine levels in FD model rats. These effects correlated with suppression of aberrant activation of the NO/cGMP/PKG signaling pathway. Together, the findings indicate that FSLAE has therapeutic potential against FD, at least in part by modulating NO/cGMP/PKG signaling. This study therefore provides preliminary scientific support for the traditional use of F. sinkiangensis leaves and identifies FSLAE as a potential novel candidate for FD management.

## 1 Introduction

Functional dyspepsia (FD) is a common functional gastrointestinal disorder defined by postprandial fullness, early satiation, nausea, vomiting, epigastric pain, bloating, and burning in the absence of an identifiable organic cause (Olson et al., 2024; He et al., 2026). Clinically, FD is categorized by predominant symptoms as postprandial distress syndrome (PDS), epigastric pain syndrome (EPS), or a mixed subtype; PDS, marked by postprandial fullness and early satiation, is the most common subtype in Asian populations (Liu et al., 2026; Kim et al., 2025). The Rome IV criteria require symptom onset at least 6 months before evaluation and fulfillment of diagnostic criteria during the preceding 3 months (Bai et al., 2025). Epidemiological estimates place adult prevalence at 10%–30%, pediatric prevalence at 3.5%–27%, and prevalence among Chinese adults at 8%–23% (Ziyang and Kuo, 2025; Yang et al., 2025). FD often follows a chronic, recurrent, and refractory course and is frequently comorbid with anxiety or depression, substantially reducing quality of life and work productivity (Ziyang and Kuo, 2025; Yang et al., 2025).

Prokinetics and proton pump inhibitors (PPIs) remain the primary treatments for functional dyspepsia (FD) in clinical practice. Prokinetics (e.g., mosapride, itopride) reduce fullness and nausea by enhancing gastrointestinal motility and accelerating gastric emptying, but their benefits are well established only for short-term use and long-term administration carries cardiovascular risks (e.g., QT interval prolongation, arrhythmia). Their effectiveness also varies across FD subgroups with different pathophysiological mechanisms, limiting their clinical applicability (Ziyang and Kuo, 2025; Yang et al., 2025). PPIs relieve acid regurgitation and heartburn by suppressing gastric acid secretion, but they offer little benefit to FD patients with normal acid output. Prolonged PPI use can cause complications such as gut microbiota dysbiosis, nutrient malabsorption, and increased infection risk, prompting widespread concern about PPI overuse (Zhou et al., 2025; Lee et al., 2025; Peng et al., 2025). This clinical picture underscores the shortcomings of current FD regimens and the need for safe, mechanism-based alternatives. Traditional ethnic medicinal plants and their bioactive constituents, which can act on multiple targets and generally have favorable safety profiles for long-term use, are therefore attracting increasing interest as potential novel therapies for FD.

*Ferula sinkiangensis* K. M. Shen is an endemic medicinal species of Ferula (Apiaceae) native to Xinjiang, China, and has been used there since at least the Tang Dynasty, when it first appeared in the Tang Materia Medica. The Pharmacopoeia of the People’s Republic of China (2025 Edition) recognizes the resin of *Ferula sinkiangensis* K. M. Shen or *Ferula fukanensis* K. M. Shen as the official botanical source of medicinal Ferula and attributes traditional effects to it including promotion of digestion, resolution of abdominal masses, relief of distention and fullness, and elimination of parasites. The digestion-promoting property is directly related to regulation of gastrointestinal function. Pharmacological research has concentrated mainly on the resin—the pharmacopoeial medicinal part—and on traditional parts such as roots and stems; these studies have confirmed activities including promotion of digestion, resolution of food retention, anti-inflammatory effects, and analgesia (Li et al., 2022; Wang et al., 2023; Dang et al., 2024). By contrast, comprehensive studies of the active constituents, pharmacological actions, and molecular mechanisms of *F. sinkiangensis* leaves are scarce. Locally, however, the leaves constitute a distinctive medicinal resource in Xinjiang ethnic medicine and have long been employed as a dietary therapy for gastrointestinal disorders to regulate bowel function, promote digestion, relieve abdominal discomfort, and improve appetite, with reported clinical and anecdotal benefits (Wariss et al., 2026). Rigorous modern scientific validation and mechanistic elucidation of the leaves’ pharmacological effects, however, remain largely lacking.

In preliminary experiments, we compared the smooth-muscle–relaxant effects of an aqueous extract of *F. sinkiangensis* leaves (FSLAE) with 30%, 50%, and 80% ethanol extracts on isolated rat small intestinal smooth muscle. All extracts produced concentration-dependent relaxation, and FSLAE showed the strongest activity, providing initial pharmacological evidence for its potential to alleviate gastrointestinal motility disorders. Building on our prior identification of systemically absorbed *F. sinkiangensis* leaf components by UPLC-Q-TOF-MS, we first constructed an interaction network between these absorbed components and FD using network pharmacology to identify putative core therapeutic targets and regulatory signaling pathways. We then established a rat model of FD by combining intragastric administration of iodoacetamide (IA) with chronic tail-clamping stress. Rats were randomly assigned to FSLAE dose groups and a positive drug control group, and gastrointestinal motility was assessed by measuring gastric emptying rate and small intestinal transit rate. We measured serum concentrations of gastrointestinal hormones and inflammatory cytokines and evaluated gastric antral histopathology by hematoxylin and eosin (H&E) staining. Finally, we quantified expression levels of key proteins in the nitric oxide/cyclic guanosine monophosphate/protein kinase G (NO/cGMP/PKG) signaling pathway by Western blot to characterize pharmacological effects and to explore a potential molecular mechanism underlying the anti-FD activity of FSLAE. This study characterizes the preliminary therapeutic efficacy, material basis, and putative mechanism by which *F. sinkiangensis* leaves exert anti-FD effects. The findings offer initial scientific support for their traditional clinical use in gastrointestinal disorders and provide a rationale for expanding the medicinal application of Ferula species to promote the high-value, sustainable development and utilization of Xinjiang’s characteristic medicinal plant resources.

## 2 Materials and methods

### 2.1 Network pharmacology analysis

Putative therapeutic targets of blood-absorbed FSLAE components identified in our previous study were retrieved from the SwissTargetPrediction database, and key pathogenic targets for FD were obtained from the GeneCards database (Yao, 2025). Overlapping targets between FSLAE absorbed components and FD were visualized with a Venn diagram. We constructed a “FSLAE blood-absorbed components–FD common targets” interaction network in Cytoscape (version 3.10.0) and selected representative absorbed components by node degree in the network combined with published literature. The protein–protein interaction (PPI) network for the common targets was retrieved from STRING and imported into Cytoscape v3.10.0 to identify hub targets of FSLAE against FD. Finally, Gene Ontology (GO) functional enrichment and Kyoto Encyclopedia of Genes and Genomes (KEGG) pathway enrichment analyses of the identified targets were performed using R (version 4.3.1).

### 2.2 Molecular docking validation

Three-dimensional structures of the core active compounds were retrieved from the PubChem Database (https://pubchem.ncbi.nlm.nih.gov/) and saved in SDF format. Ligand structures were preprocessed in AutoDock Tools 1.5.6: salt ions were removed, polar hydrogens were added, Gasteiger partial charges were computed, and rotatable bonds were defined. Crystal structures of the target proteins endothelial nitric oxide synthase (eNOS, PDB ID: 4D1P) and protein kinase G 1 (PKG1, PDB ID: 6BG2) were obtained from the RCSB Protein Data Bank (RCSB PDB, http://www.rcsb.org/) and saved in PDB format. Water molecules and native ligands were removed from the protein files using PyMOL, and the cleaned structures were re-saved in PDB format; binding-pocket parameters were determined with the GetBox plugin. Processed protein and ligand files were imported separately into AutoDock Tools (ADT) version 1.5.6 and converted to PDBQT format. Molecular docking simulations were performed with AutoDock Vina version 1.1.2, and docking poses were visualized with PyMOL (version 2.6.0).

### 2.3 Plant materials and extraction procedure

Leaves of *F. sinkiangensis* were collected in Ili Kazakh Autonomous Prefecture, Xinjiang, China, and authenticated as the dried leaves of *F. sinkiangensis* by Chief Pharmacist Li Yonghe (Department of Pharmacy, Xinjiang Autonomous Region Hospital of Traditional Chinese Medicine). Two hundred grams of *F. sinkiangensis* leaf decoction pieces were pulverized, passed through a 40-mesh sieve, and mixed with ultrapure water at a ratio of 10:1 (water:material, w/w). The mixture was soaked for 1 h, extracted in a water bath at 80 °C for 1.5 h, and filtered while hot. The extraction was repeated three times, and the resulting filtrates were combined. The combined filtrate was concentrated under reduced pressure in a water bath at 65 °C to yield a fluid extract with a crude drug concentration of 2 g/mL, which was stored at −20 °C. The extract was sealed in ampoules prior to use in the animal experiments of this study.

### 2.4 Experimental animals

Sixty male Sprague–Dawley (SD) rats of specific pathogen–free (SPF) grade, aged 7–8 weeks and weighing 180–220 g, were supplied by the Laboratory Animal Center of Xinjiang Medical University (Laboratory Animal Use Permit No. SYXK (Xin) 2023-0004). After a 1 week acclimation period, rats were enrolled in experiments only after stable vital signs and normal weight gain were confirmed. Animals were housed in the Animal Center’s barrier facility under controlled conditions: ambient temperature 22 °C–26 °C, relative humidity 40%–70%, and a 12 h/12 h light–dark cycle, with *ad libitum* access to standard chow and sterile water. The experimental protocol was approved by the Institutional Animal Care and Use Committee (IACUC) of Xinjiang Medical University (IACUC Approval No. XJMU-IACUC-20240320-25), and all procedures followed the Guidelines for Ethical Review of Laboratory Animal Welfare, relevant laboratory animal management regulations, and the 3 R principles (Reduction, Refinement, Replacement). After an adaptive feeding period, rats were randomized into a blank control group and a modeling group using a random number table. Rats in the blank control group received 0.2 mL of 2% sucrose solution by gavage once daily for six consecutive days while maintained on standard care. Rats in the modeling group were gavaged with 0.2 mL of 2% sucrose solution containing 0.1% iodoacetamide (IA) once daily for six consecutive days. Beginning on day 7, the modeling group underwent chronic tail-clamping stress: the distal one-third of the tail was clamped with long sponge forceps for 30 min per session, four sessions per day at 3-h intervals, for seven consecutive days. Successful modeling was defined by the following criteria: listlessness, dry and dull fur, loose stools, markedly reduced food intake and spontaneous activity, significantly decreased gastric emptying rate and small intestinal transit rate relative to the blank group, and absence of obvious organic pathological changes in gastric antrum tissue. After successful modeling, the rats were randomly assigned to five groups of 10 animals each by a random number table: model control, high-dose FSLAE, medium-dose FSLAE, low-dose FSLAE, and mosapride (Lunan Beite Pharmaceutical Co., Ltd., Linyi, China, Lot No. 26251223) positive control. Drug treatment was given once daily by oral gavage for seven consecutive days. Rats in the blank and model control groups received an equal volume of normal saline by gavage. Rats in the remaining groups received their respective test drugs by the same route. The high-, medium-, and low-dose FSLAE groups received 150, 100, and 50 mg/kg, respectively (doses calculated on the crude drug content of the fluid extract); this gradient was chosen with reference to the literature and validated by pharmacodynamic results from our preliminary experiments. The mosapride positive-control group received 1.35 mg/kg, the dose equivalent to the clinical regimen for a 60 kg adult. All animal doses were calculated using the human-to-rat body-surface-area conversion coefficient.

### 2.5 Determination of gastric emptying and small intestinal transit function

Six rats were randomly selected from each group, fasted with *ad libitum* access to water for 24 h, and then given 2 mL of black semi-solid nutrient paste by intragastric gavage (paste prepared from 2 g activated carbon powder, 8 g soluble starch, 8 g sucrose, 10 g sodium carboxymethyl cellulose (CMC-Na), 16 g milk powder, and 250 mL distilled water). After administration, the animals were anesthetized and subjected to laparotomy. The intact stomach was removed and weighed, then opened along the greater curvature, rinsed with normal saline, blotted with filter paper, and reweighed to determine the gastric emptying rate. The mesentery was exposed, and the small intestine from the gastric pylorus to the ileocecal junction was excised and laid flat without stretching. The distance the semi-solid paste migrated from the pylorus and the total length of the small intestine were measured to calculate the small intestinal transit rate.

### 2.6 Histopathological observation of gastric tissue

Gastric antrum tissues were collected from rats in each group, fixed in 4% paraformaldehyde, dehydrated through graded ethanol, embedded in paraffin, and serially sectioned. After dewaxing and rehydration, sections were stained with a HE High-Definition Constant Staining Kit (Wuhan Servicebio Technology Co., Ltd., Wuhan, China, Lot No. G1076). Histopathological features of the gastric tissue were examined and photographed by light microscopy.

### 2.7 Enzyme-linked immunosorbent assay (ELISA) detection

After the final administration, rats were fasted for 24 h with free access to water, and blood was collected from the abdominal aorta under 10% chloral hydrate anesthesia. Whole blood was allowed to clot at room temperature for 30 min, then centrifuged at 3000 rpm for 15 min at 4 °C to obtain serum. Serum samples were aliquoted into sterile cryotubes and stored at −80 °C to prevent repeated freeze–thaw cycles. All enzyme-linked immunosorbent assay (ELISA) measurements were performed by investigators blinded to treatment group assignments. Serum levels of ghrelin (Shanghai Jianglai Biotechnology Co., Ltd., Shanghai, China, Lot No. JL18312), motilin (MTL, Shanghai Jianglai Biotechnology Co., Ltd., Shanghai, China, Lot No. JL12355), gastrin (Gas, Shanghai Jianglai Biotechnology Co., Ltd., Shanghai, China, Lot No. JL21322), somatostatin (SS, Shanghai Jianglai Biotechnology Co., Ltd., Shanghai, China, Lot No. JL12919), tumor necrosis factor-α (TNF-α, Shanghai Jianglai Biotechnology Co., Ltd., Shanghai, China, Lot No. JL13202), interleukin-17A (IL-17A, Shanghai Jianglai Biotechnology Co., Ltd., Shanghai, China, Lot No. JL20880), interleukin-6 (IL-6, Shanghai Jianglai Biotechnology Co., Ltd., Shanghai, China, Lot No. JL20896), interleukin-4 (IL-4, Shanghai Jianglai Biotechnology Co., Ltd., Shanghai, China, Lot No. JL20894), nitric oxide (NO, Shanghai Jianglai Biotechnology Co., Ltd., Shanghai, China, Lot No. JL14567), and cyclic guanosine monophosphate (cGMP, Shanghai Jianglai Biotechnology Co., Ltd., Shanghai, China, Lot No. JL11179) were quantified using commercial ELISA kits according to the manufacturers’ protocols. Each experimental group comprised three independent biological replicates (individual rats), and each serum sample was measured in technical duplicate to ensure accuracy. The mean absorbance from the duplicates was used for all subsequent statistical analyses.

### 2.8 Western blotting analyses

Total protein was extracted from the gastric antrum tissues of rats in each group. After protein concentration quantification using the bicinchoninic acid (BCA) assay, equal amounts of protein samples were separated by sodium dodecyl sulfate–polyacrylamide gel electrophoresis (SDS-PAGE), electrotransferred onto polyvinylidene fluoride (PVDF) membranes, and then blocked with blocking buffer at room temperature. The membranes were incubated overnight at 4 °C with primary antibodies against endothelial nitric oxide synthase (eNOS, Abcam plc, Cambridge, United Kingdom, Lot No. ab199956), phospho-endothelial nitric oxide synthase (p-eNOS, Abcam plc, Cambridge, United Kingdom, Lot No. ab184154), and protein kinase G 1 (PKG1, Abcam plc, Cambridge, United Kingdom, Lot No. ab110124) at a dilution of 1:1000, as well as the β-actin internal reference antibody (Abcam plc, Cambridge, United Kingdom, Lot No. ab8227) at a dilution of 1:10000, all following the manufacturers’ instructions. Subsequently, the membranes were incubated with the corresponding horseradish peroxidase (HRP)-conjugated secondary antibody on an orbital shaker at room temperature for 2 h. Protein bands were visualized using an enhanced chemiluminescence (ECL) reagent and imaged with a gel imaging system. Densitometric analysis of Western blot bands was performed with ImageJ. Each target protein’s relative expression was calculated as the ratio of its band grayscale value to the β-actin internal reference band on the same membrane. For phosphorylated proteins, phosphorylation levels were normalized to the corresponding total protein.

### 2.9 Statistical analysis

SPSS 24.0 statistical software was used for analysis. The metric data that conforms to a normal distribution is represented by mean ± standard deviation. When both the homogeneity of variance and normality of the measurement data are met, one-way ANOVA is used for multi group comparison, and LSD-t test is used for inter group comparison; When the measurement data does not meet the homogeneity of variance test or normality test, non parametric rank sum test is used. The inspection level is α = 0.05. When *P* < 0.05, it is considered statistically significant, and a statistical graph is drawn using GraphPad Prism 8.

## 3 Results and discussion

### 3.1 Blood-absorbed components of FSLAE

In our previous study, UPLC-Q-TOF-MS was used to systematically identify and characterize the blood-absorbed components of FSLAE following intragastric administration in rats. We identified and confirmed a total of 23 absorbed prototype components in the blood (detailed information on these 23 prototype components is provided in the Supplementary Material 1). These components were predominantly phenolic acids and flavonoids and also included other structural classes such as alkaloids and coumarins. Detailed information for each blood-absorbed component is provided in Table 1.

**TABLE 1:** Information of blood-absorbed components of FSLAE.

No.	Compound name	Molecular formula	Chemical class
1	D-sorbitol	C_6_H_14_O_6_	Sugar alcohols
2	5,6,2′,3′,6′-pentamethoxyflavone	C_20_H_20_O_7_	Flavonoids
3	Turanose	C_12_H_22_O_11_	Disaccharides
4	2-Isopropylmalic acid	C_7_H_12_O_5_	Organic acids
5	3-p-Coumaroylquinic acid	C_16_H_18_O_8_	Phenolic acids
6	7-Hydroxycoumarin sulfate ester	C_9_H_6_O_6_S	Coumarins
7	3-O-Feruloylquinic acid	C_17_H_20_O_9_	Phenolic acids
8	Isoferulic acid	C_10_H_10_O_4_	Phenolic acids
9	Aesculetin	C_9_H_6_O_4_	Coumarins
10	Geraniol	C_10_H_18_O	Monoterpenes
11	Anisic acid	C_8_H_8_O_3_	Phenolic acids
12	4-Methylumbelliferone	C_10_H_8_O_3_	Coumarins
13	Ferulic acid	C_10_H_10_O_4_	Phenolic acids
14	Luteolin 4′-glucoside	C_21_H_20_O_11_	Flavonoid glycosides
15	Scopoletin	C_10_H_8_O_4_	Coumarins
16	Cinnamic acid	C_9_H_8_O_2_	Phenolic acids
17	2-Methylbutyl (acetyloxy)acetate	C_9_H_16_O_4_	Esters
18	Traumatic acid	C_12_H_20_O_4_	Fatty acids
19	3,16-Dihydroxypalmitic acid	C_16_H_32_O_4_	Fatty acids
20	4-Methoxy-5-nonylbenzene-1,3-diol	C_16_H_26_O_3_	Phenols
21	Pulchelloid D	C_25_H_34_O_8_	Sesquiterpenes
22	Citronellyl acetate	C_12_H_22_O_2_	Monoterpene esters
23	α-iso-cubebenol	C_15_H_24_O	Sesquiterpenes

### 3.2 Network pharmacology analysis

**FIGURE 1:**
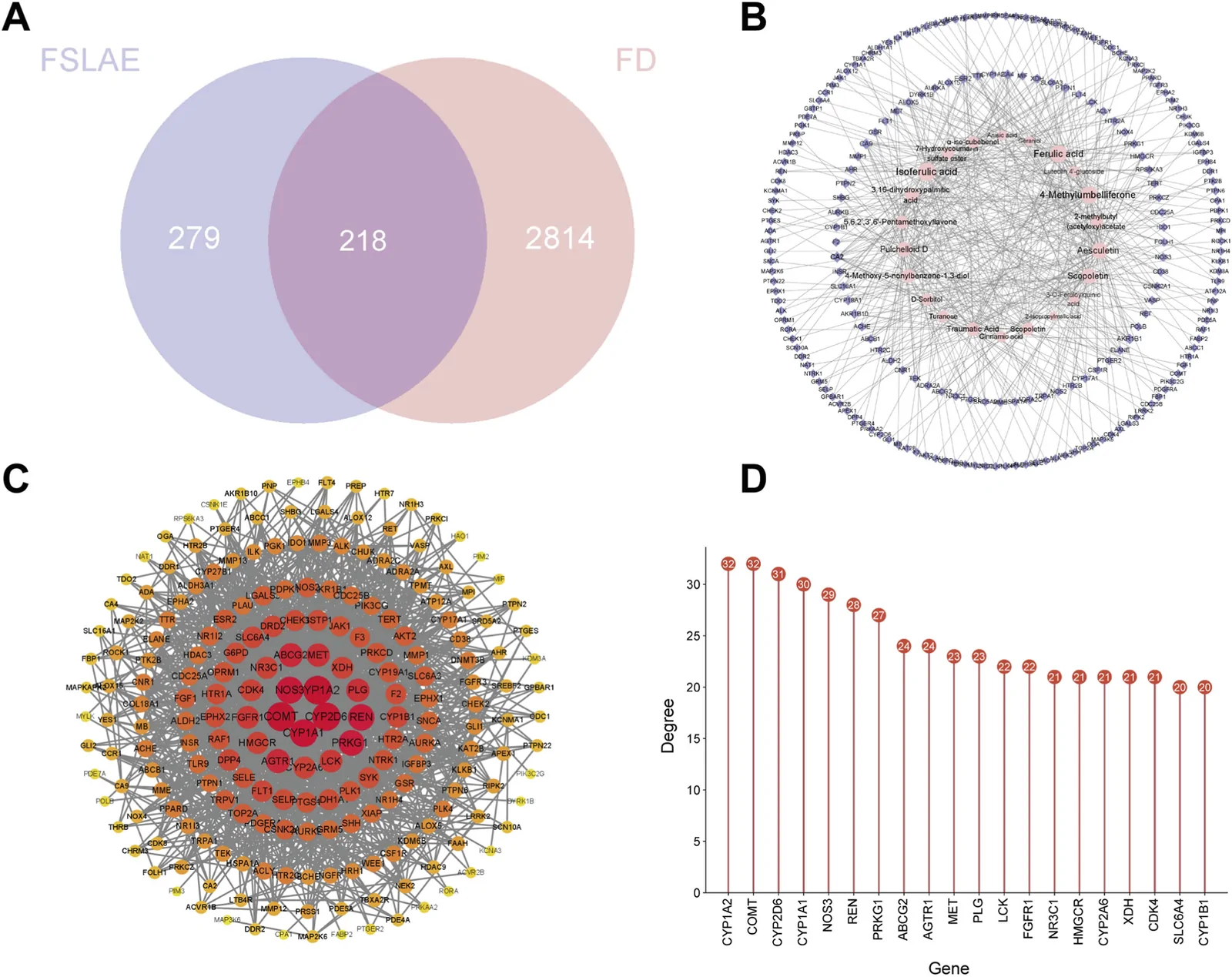
Screening and analysis of core targets for bioavailable components of FSLAE and action targets of FD. **(A)** Venn diagram of FSLAE plasma-absorbed components and FD action targets. **(B)** “FSLAE plasma-absorbed components - FD common targets” network diagram. **(C)** Protein-Protein Interaction (PPI) network diagram of the intersection targets. **(D)** Statistical chart of Degree values of core targets.

We retrieved and screened public databases to obtain 497 putative therapeutic targets linked to blood-absorbed components of FSLAE and 3032 FD-associated pathogenic targets. Intersection analysis of these sets yielded 218 shared putative targets of FSLAE blood-absorbed components against FD (Figure 1A). Using these common targets, we constructed a “FSLAE blood-absorbed components–FD common targets” interaction network (Figure 1B). Network topology analysis, using Degree as the primary index, identified the core active blood-absorbed components of FSLAE against FD; isoferulic acid, ferulic acid, 4-methylumbelliferone, and aesculetin exhibited the highest node degree values. Their detailed topological parameters are listed in Table 2. The protein–protein interaction (PPI) network is shown in Figure 1C. Further Degree-based screening revealed the hub targets of FSLAE against FD: CYP1A2, COMT, CYP2D6, CYP1A1, NOS3, REN, PRKG1, and ABCG2. The Degree values for these hub targets are reported in Figure 1D.

**TABLE 2:** Core components of FSLAE in the treatment of FD.

No.	Component	PubChem CID	CAS	Degree
1	Isoferulic acid	736186	537-73-5	50
2	Ferulic acid	445858	1135-24-6	46
3	4-Methylumbelliferone	5280567	90-33-5	45
4	Aesculetin	5281416	305-01-1	39
5	Scopoletin	5280460	92-61-5	35
6	Pulchelloid D	/	130223-09-5	30
7	3,16-Dihydroxypalmitic acid	56927872	/	26
8	4-Methoxy-5-nonylbenzene-1,3-diol	11277128	/	26
9	5,6,2′,3′,6′-pentamethoxyflavone	21580516	/	24
10	Traumatic acid	5283028	6402-36-4	24

**FIGURE 2:**
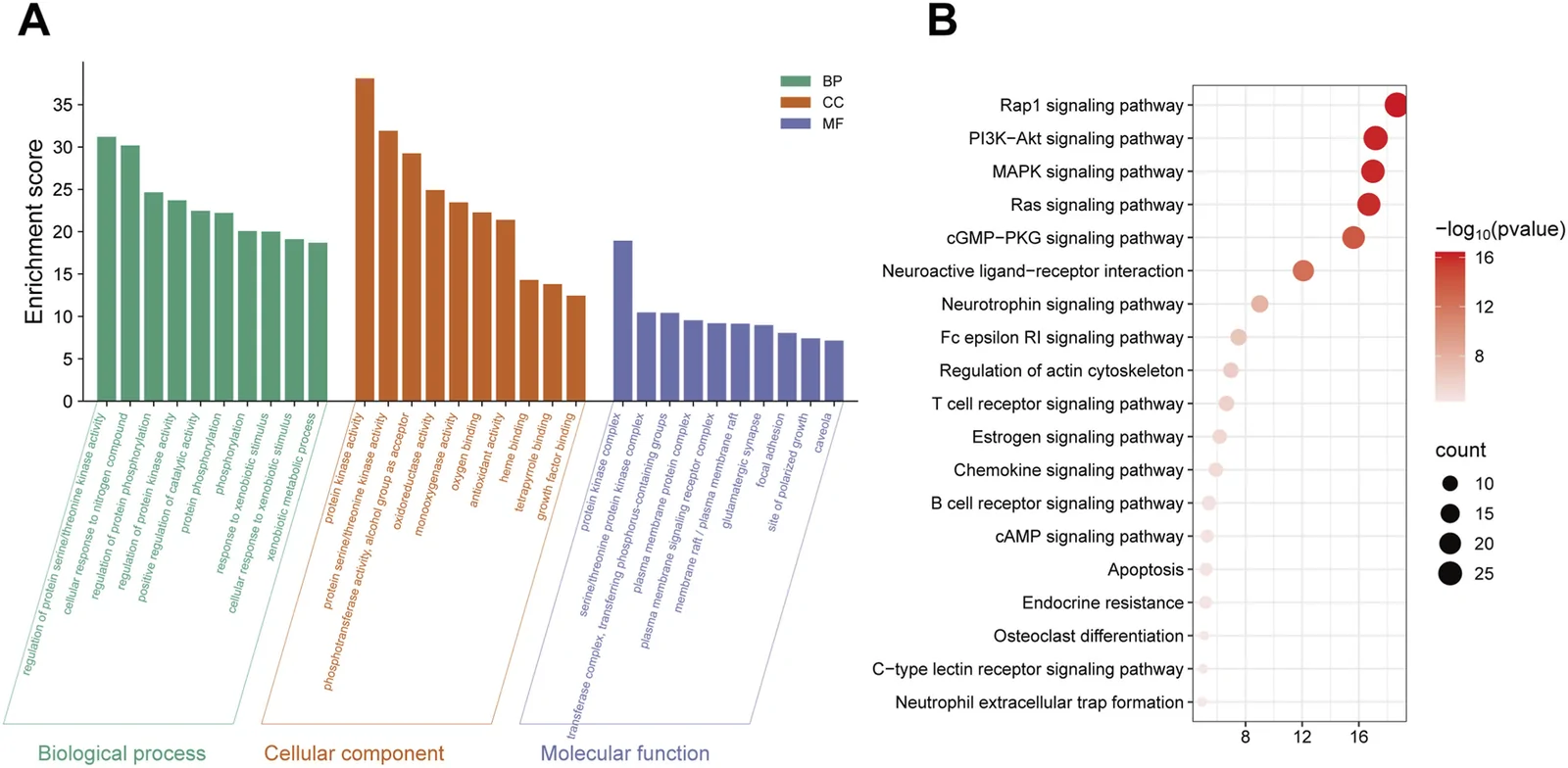
Enrichment analysis results. **(A)** GO biological enrichment analysis. **(B)** KEGG pathway enrichment analysis.

GO and KEGG pathway enrichment analyses were conducted on the 218 intersection targets shared by the blood-absorbed components of FSLAE and FD (Figure 2). GO enrichment highlighted core biological features concentrated in biological processes such as regulation of protein serine/threonine kinase activity, protein phosphorylation, and responses to oxidative stress and xenobiotic stimulus; cellular components including the protein kinase complex, plasma membrane signaling receptor complex, and membrane raft; and molecular functions such as protein kinase binding, oxidoreductase activity, and antioxidant activity. These enriched terms align closely with key pathological aspects of FD, including impaired gastrointestinal motility, dysregulated inflammatory responses, and disrupted oxidative stress balance.

KEGG pathway enrichment identified core regulatory pathways through which FSLAE acts against FD, including Rap1, PI3K-Akt, MAPK, Ras, and NO/cGMP/PKG signaling pathways, with NO/cGMP/PKG designated as the primary validated target in this study. The enrichment also highlighted pathways implicated in brain–gut axis dysfunction and low-grade gastrointestinal inflammation, such as neuroactive ligand–receptor interaction and T/B cell receptor signaling. These findings indicate that FSLAE exerts therapeutic effects on FD via multi-component, multi-target, and multi-pathway mechanisms, likely involving regulation of gastrointestinal motility signaling, suppression of inflammation and oxidative stress, and modulation of brain–gut axis function, thereby providing systematic bioinformatic support for subsequent *in vivo* pharmacodynamic and mechanistic validation experiments.

### 3.3 Molecular docking validation

Network pharmacology indicated that FSLAE may act on FD through multiple components, targets, and pathways. It highlighted several signaling cascades implicated in FD progression, including Rap1, PI3K-Akt, and NO/cGMP/PKG, and identified putative hub targets such as CYP1A2, NOS3, and PRKG1. Impaired gastrointestinal motility is the principal clinical pathophysiological feature of FD and remains the primary focus of current pharmacological interventions. The NO/cGMP/PKG pathway is a central regulator of gastrointestinal smooth muscle contraction and relaxation and thus of motility homeostasis; its dysregulation is closely linked to delayed gastric emptying in FD models. Consequently, we concentrated on this pathway to investigate the molecular basis of FSLAE’s therapeutic effects. We selected eNOS and PKG1, encoded by the upstream and downstream hub targets NOS3 and PRKG1, We used molecular docking to predict ligand–protein interactions for the five putative systemically absorbed active compounds identified by network pharmacology (isoferulic acid, ferulic acid, 4-methylumbelliferone, aesculetin, and scopoletin). A binding energy ≤ −5.0 kcal/mol served as the threshold for favorable ligand–receptor binding, a widely accepted cutoff in molecular docking studies of natural product–target interactions (Ren et al., 2024).

**FIGURE 3:**
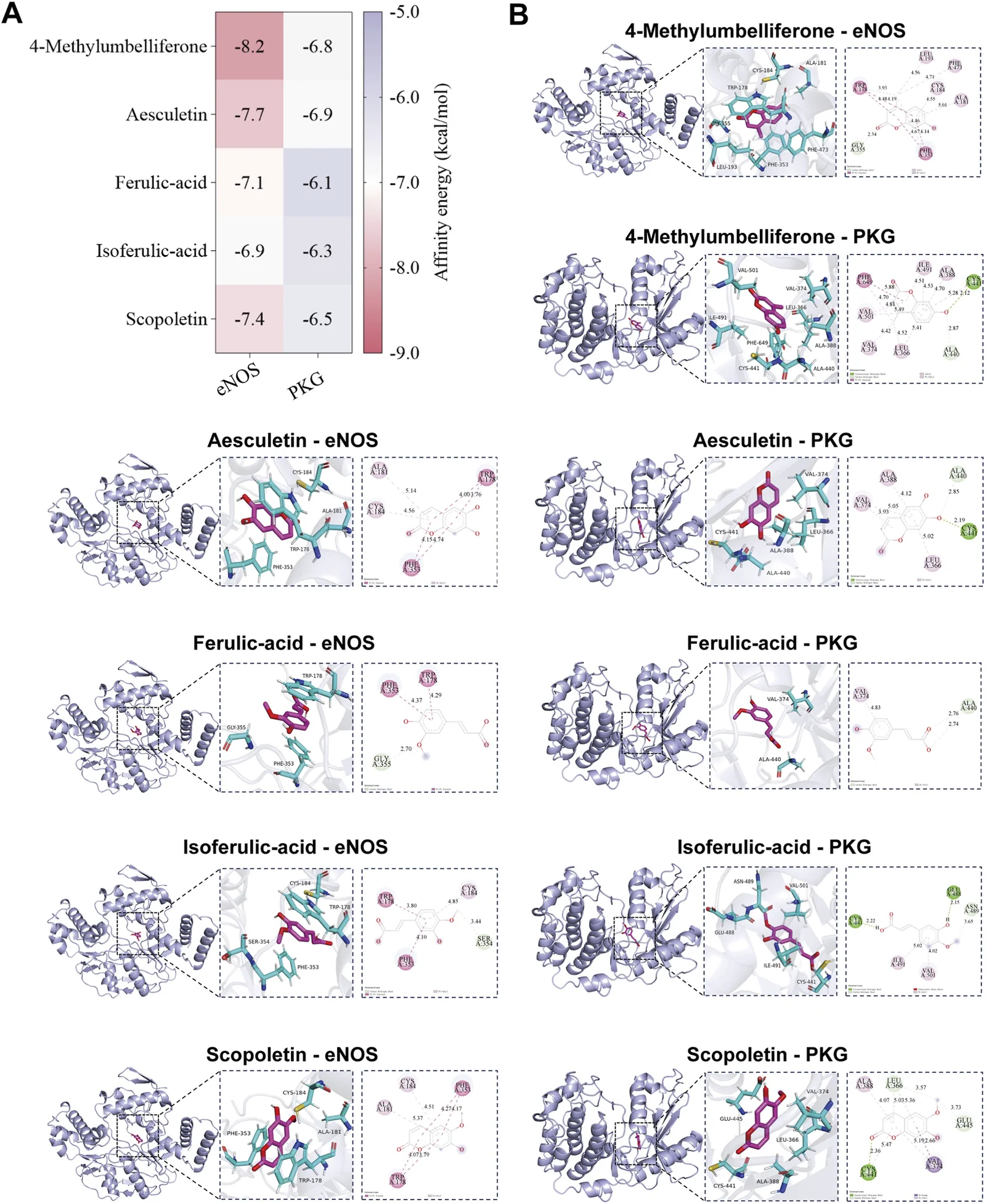
Molecular docking results between the core active ingredients and eNOS and PKG proteins. **(A)** Heatmap of binding affinities between the core active ingredients and the eNOS and PKG proteins. Values are binding energies (unit: kcal/mol); larger absolute binding-energy values indicate stronger ligand–receptor affinity. **(B)** Visualization of ligand–target binding. From left to right: three-dimensional (3D) structure of the ligand–receptor complex, ligand conformation and interactions with amino acid residues in the binding pocket, and the two-dimensional (2D) map of ligand–residue interactions.

The molecular docking results are presented in Figure 3. All five putative core active ingredients bound to both eNOS and PKG1 with binding energies below −5.0 kcal/mol, suggesting the potential for stable intermolecular interactions. 4-Methylumbelliferone exhibited the strongest predicted binding affinity for eNOS (binding energy = −8.2 kcal/mol), and aesculetin exhibited the highest predicted binding affinity for PKG1 (binding energy = −6.9 kcal/mol). Docking visualizations suggested that these ligands may occupy the active pocket regions of eNOS and PKG1 and form hydrogen bonds and hydrophobic interactions that may stabilize the ligand-receptor complexes. These computational results suggest that the putative core systemically absorbed ingredients of FSLAE may directly interact with key nodes of the NO/cGMP/PKG signaling pathway at the molecular level, providing a preliminary theoretical basis and predictive guidance for subsequent experimental validation of how FSLAE may modulate this pathway to ameliorate FD-like symptoms.

### 3.4 Comparison of general condition, body weight changes and gastrointestinal motility indexes among groups

#### 3.4.1 Observation of general condition of rats

After drug treatment, rats in the blank control group exhibited dense, smooth, glossy fur, an alert mental state, and normal spontaneous activity, indicating stable physiology and good nutritional status. Rats in the model control group appeared listless, showed reduced spontaneous activity, and had sparse, dry, rough, and dull fur, reflecting markedly poorer growth and nutrition than the blank controls. Each FSLAE dose group showed varying improvements in mental state, activity, and fur condition compared with the model controls, with noticeable recovery of fur texture and gloss. The high-dose FSLAE group demonstrated the greatest improvement, and its overall condition most closely resembled that of the blank control group.

#### 3.4.2 Changes of body weight in rats

**FIGURE 4:**
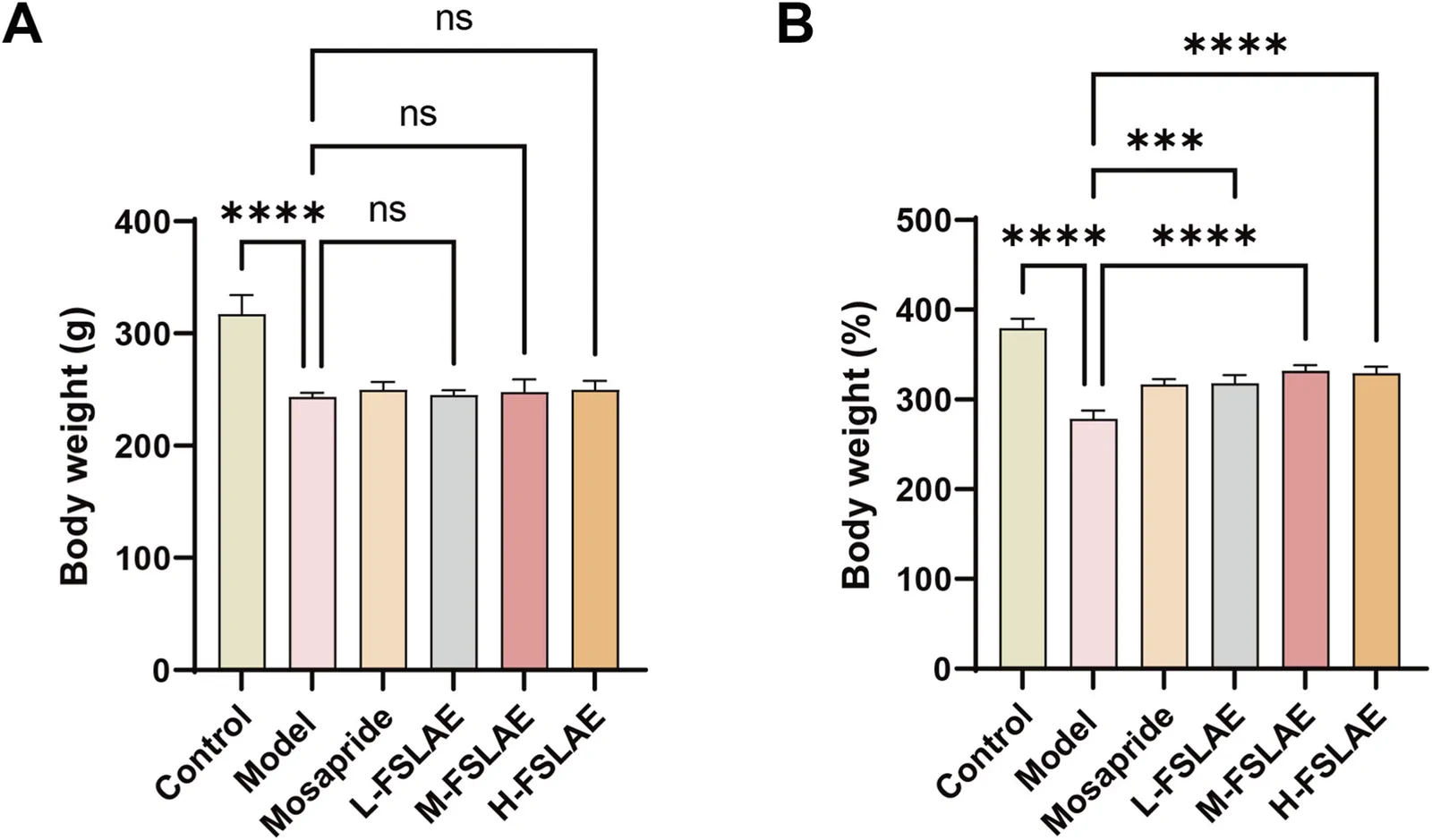
Comparative Analysis of Body Weight in Each Group of Rats Post-Modeling and Post-Administration Intervention (‾X ± s, n = 10). **(A)** Body weight of rats in each group after modeling. **(B)** Body weight of rats in each group after administration. *P < 0.05, **P < 0.01, ***P < 0.001, ****P < 0.0001; ns, no significant difference.

Before treatment, compared with the blank control group, the model control group and all administration groups exhibited a markedly slower body weight gain and a significantly lower body weight (*P* < 0.0001), indicating that the IA plus tail-clamping protocol substantially inhibited normal weight gain in rats and met the core criteria for successful modeling (Figure 4A). After drug intervention, the model control group still showed a pronounced lag in weight gain and a persistently reduced body weight compared with the blank control group (*P* < 0.0001). Relative to the model control group, all FSLAE dose groups and the positive control group demonstrated a significant increase in body weight and an improved weight-gain rate, with highly significant differences (*P* < 0.001, *P* < 0.0001), indicating that each treatment regimen effectively reversed the modeling-induced inhibition of weight gain in rats (Figure 4B).

#### 3.4.3 Changes of gastrointestinal motility indexes in rats

**FIGURE 5:**
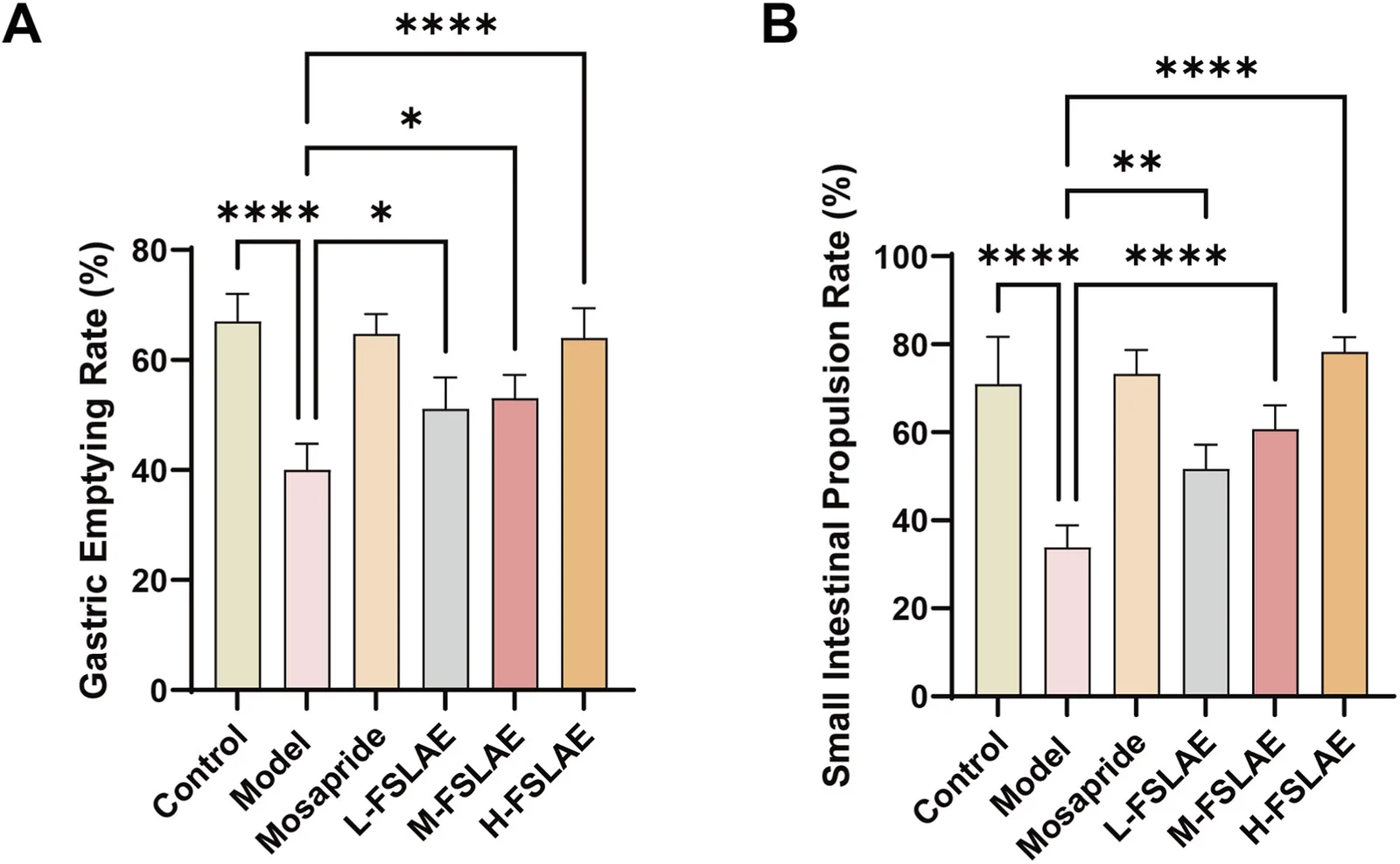
Effects of FSLAE on gastric emptying rate and small intestinal transit rate in FD model rats (‾X ± s, n = 10). **(A)** Gastric emptying rate of rats in each group. **(B)** Small intestinal transit rate of rats in each group. *P < 0.05, **P < 0.01, ***P < 0.001, ****P < 0.0001; ns, no significant difference.

Gastric emptying measurements (Figure 5A) showed that the model control group had a markedly reduced gastric emptying rate versus the blank control group (*P* < 0.0001), consistent with the core pathophysiology of gastrointestinal motility disorder in FD. Each FSLAE dose significantly increased gastric emptying relative to the model control (*P* < 0.05), and the high-dose FSLAE group produced the largest improvement with an extremely significant effect (*P* < 0.0001). Small intestinal transit measurements (Figure 5B) similarly showed a sharply reduced transit rate in the model control group compared with the blank control group (*P* < 0.0001). All FSLAE dose groups produced highly significant increases in small intestinal transit relative to the model control (*P* < 0.01), with the high-dose FSLAE group yielding the greatest improvement (*P* < 0.0001).

### 3.5 Pathomorphological observation of gastric tissues by HE staining

**FIGURE 6:**
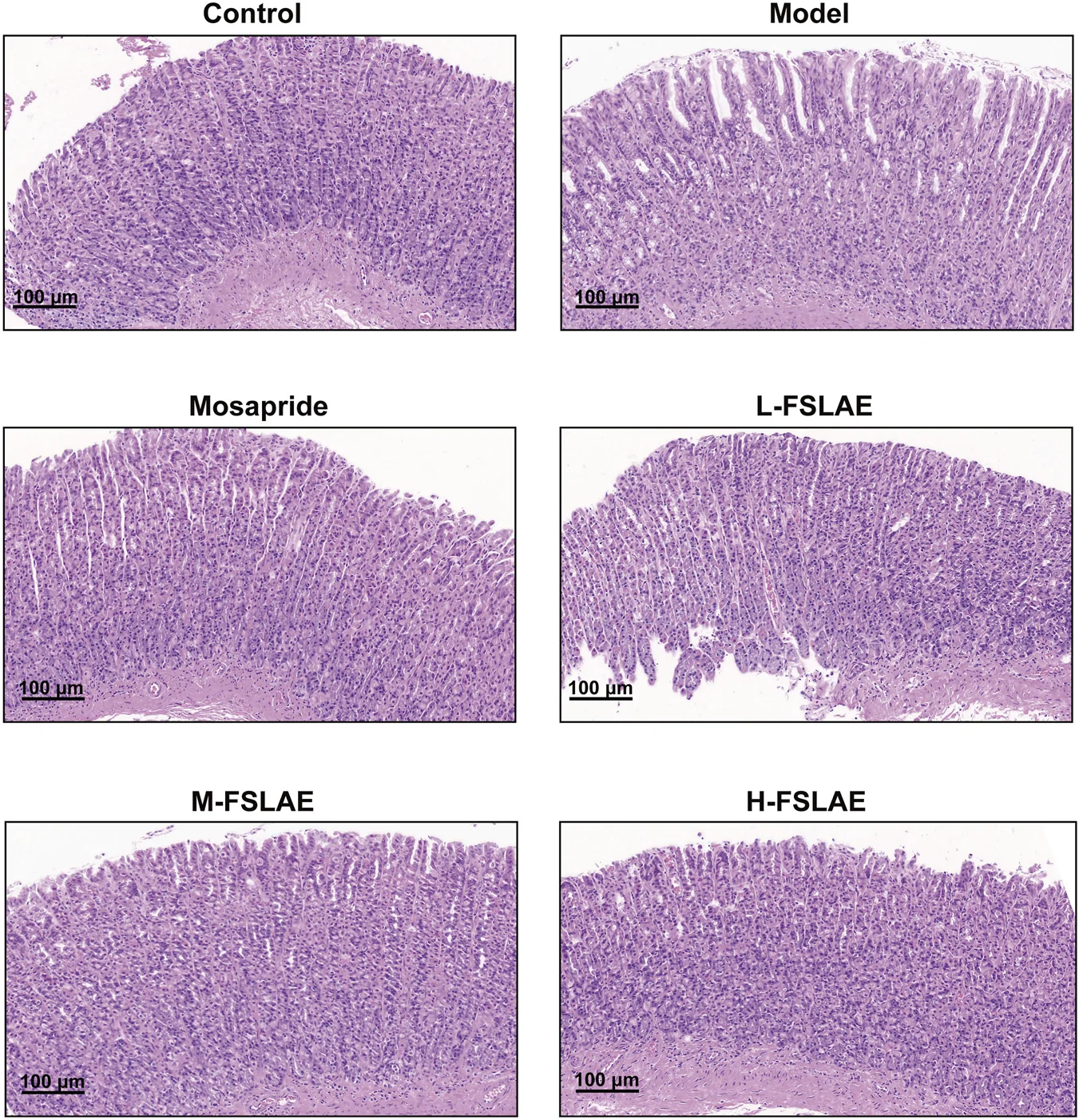
Effects of FSLAE on gastric mucosal injury in FD model rats.

At the end of the treatment period, H&E staining (Figure 6) showed that gastric antral tissues in all groups retained structural integrity with continuous mucosal layers. No erosions, ulcers, necrotic foci, or notable organic pathological changes—such as inflammatory cell infiltration, hemorrhage, or edema—were observed. This finding aligns with the defining feature of FD, namely the absence of organic damage. Compared with the normal control group, the model control group exhibited only slight loosening of glandular arrangement and a minor reduction in structural regularity in limited regions of the gastric antrum. In contrast, the FSLAE dose groups showed no additional pathological changes, and their glandular arrangement and mucosal morphology remained comparable to those of the normal control group.

### 3.6 Effects of FSLAE on serum gastrointestinal hormone levels in FD model rats

**FIGURE 7:**
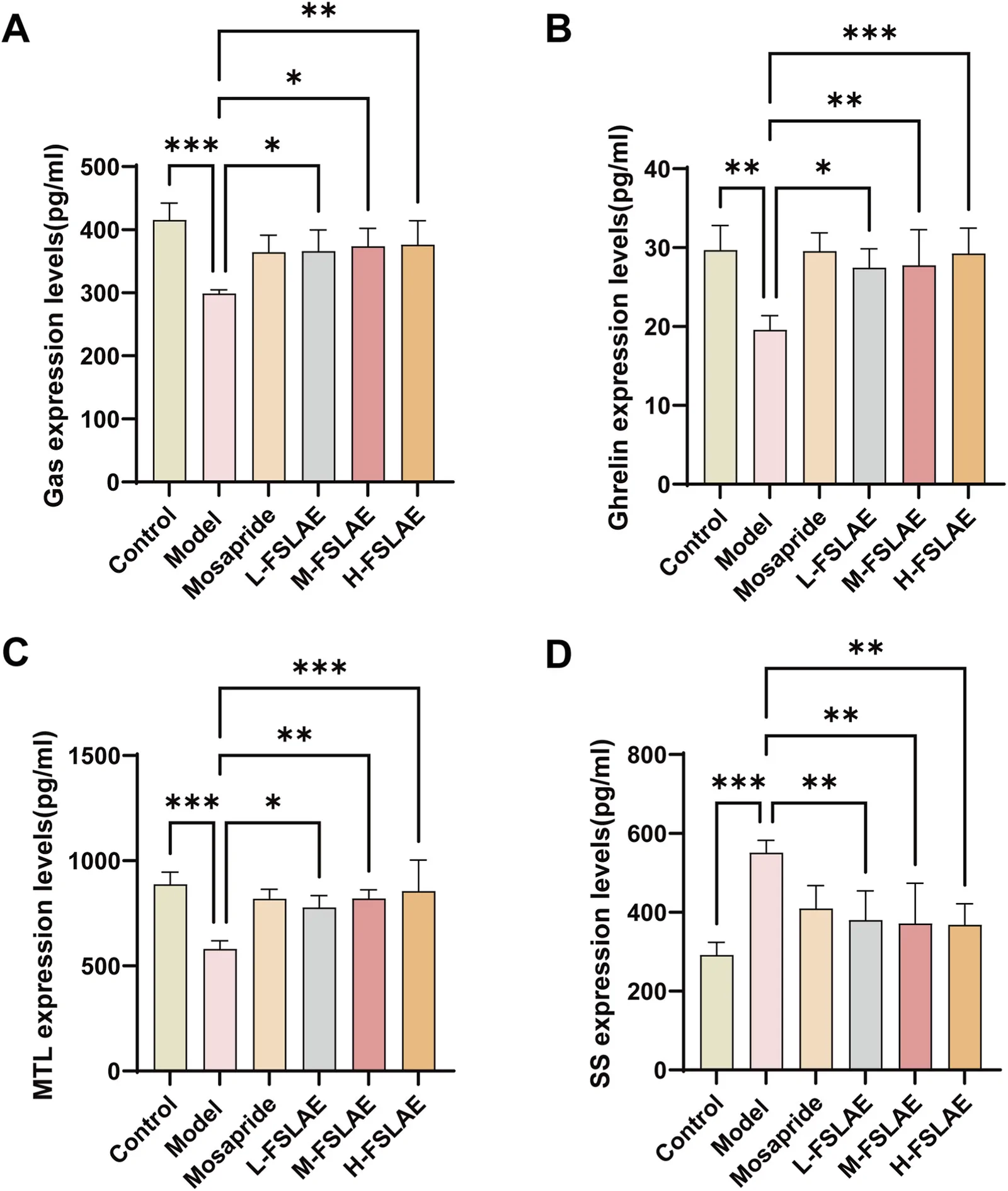
Effects of FSLAE on serum gastrointestinal hormone levels in FD model rats (‾X ± s, n = 10). **(A)** Serum GAS levels. **(B)** Serum Ghrelin levels. **(C)** Serum MTL levels. **(D)** Serum SS levels. *P < 0.05, **P < 0.01, ***P < 0.001, ****P < 0.0001.

Serum gastrointestinal hormones in rats were measured by ELISA; the results appear in Figure 7. Relative to the blank control group, the model control group showed markedly lower serum concentrations of GAS, MTL, and Ghrelin (*P* < 0.01), while SS was markedly higher (*P* < 0.001), indicating pronounced dysregulation of gastrointestinal hormone secretion in the FD model rats. Compared with the model control group, each FSLAE dose significantly ameliorated these hormonal disturbances: GAS, MTL, and ghrelin increased in a dose-dependent fashion (*P* < 0.05), with the high-dose FSLAE group showing the largest effect, while SS decreased significantly (*P* < 0.01). These findings indicate that FSLAE dose-dependently restored gastrointestinal hormone balance in FD model rats.

### 3.7 Effects of FSLAE on serum inflammatory cytokine levels in FD model rats

**FIGURE 8:**
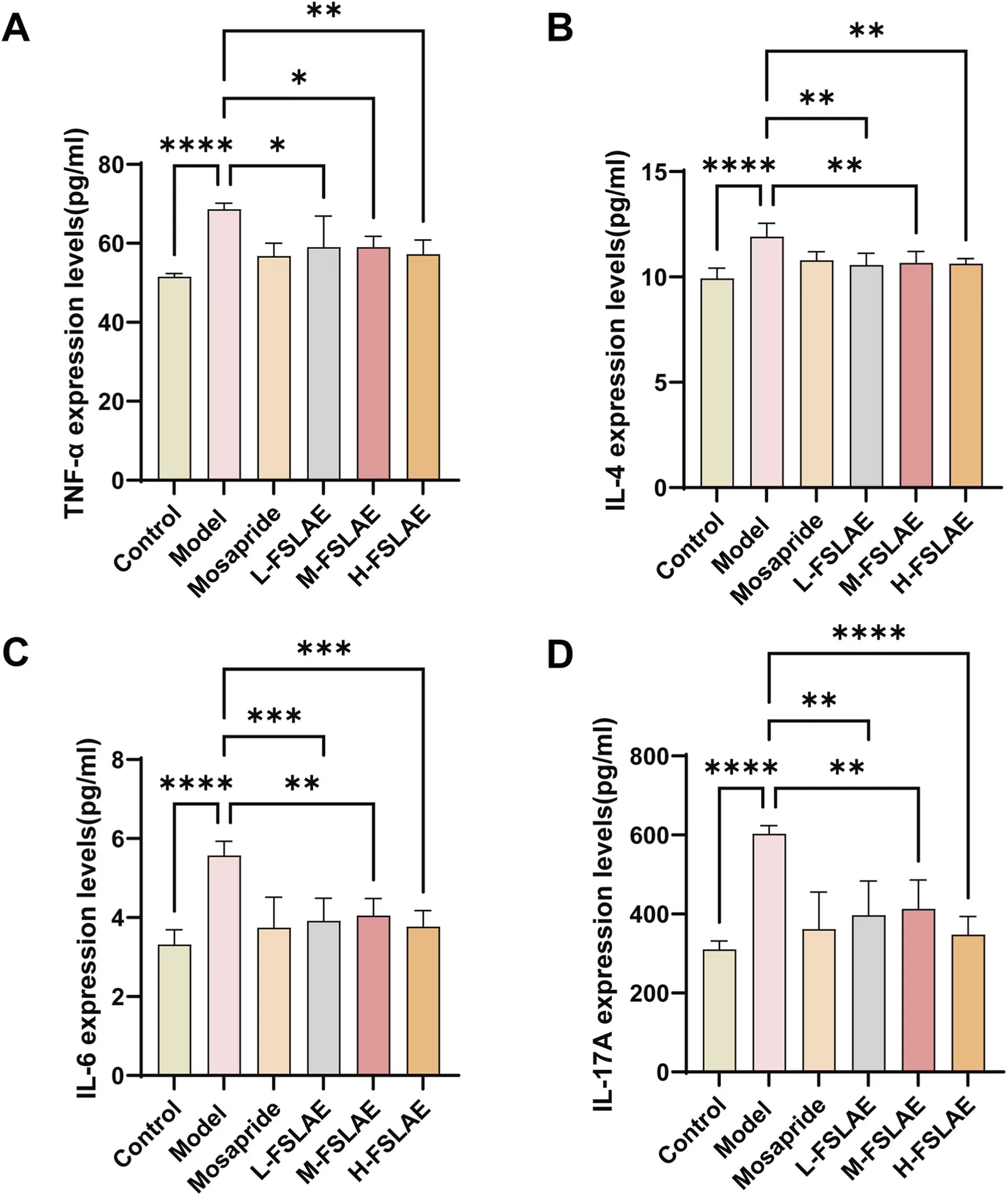
Effects of FSLAE on serum inflammatory cytokine levels in FD model rats (‾X ± s, n = 10). **(A)** Serum TNF-α levels; **(B)** Serum IL-4 levels; **(C)** Serum IL-6 levels; **(D)** Serum IL-17A levels. *P < 0.05, **P < 0.01, ***P < 0.001, ****P < 0.0001.

Serum inflammatory cytokines in rats were measured by ELISA assay; the results are shown in Figure 8. Relative to the blank control group, serum levels of TNF-α, IL-4, IL-17A, and IL-6 were markedly elevated in the model control group (*P* < 0.0001), indicating pronounced gastrointestinal inflammation in the FD model rats. Compared with the model control group, all doses of FSLAE significantly reduced these cytokine levels in a dose-dependent manner. TNF-α was significantly decreased (*P* < 0.05, *P* < 0.01), IL-4 was markedly decreased (*P* < 0.01), IL-6 was markedly decreased (*P* < 0.01, *P* < 0.001), and IL-17A was markedly decreased (*P* < 0.01, *P* < 0.0001). The high-dose FSLAE group produced the strongest inhibition of all four cytokines. These findings indicate that FSLAE effectively attenuates the gastrointestinal inflammatory response in FD model rats by lowering serum inflammatory cytokine levels.

### 3.8 Effects of FSLAE on serum cGMP and NO levels in FD model rats

**FIGURE 9:**
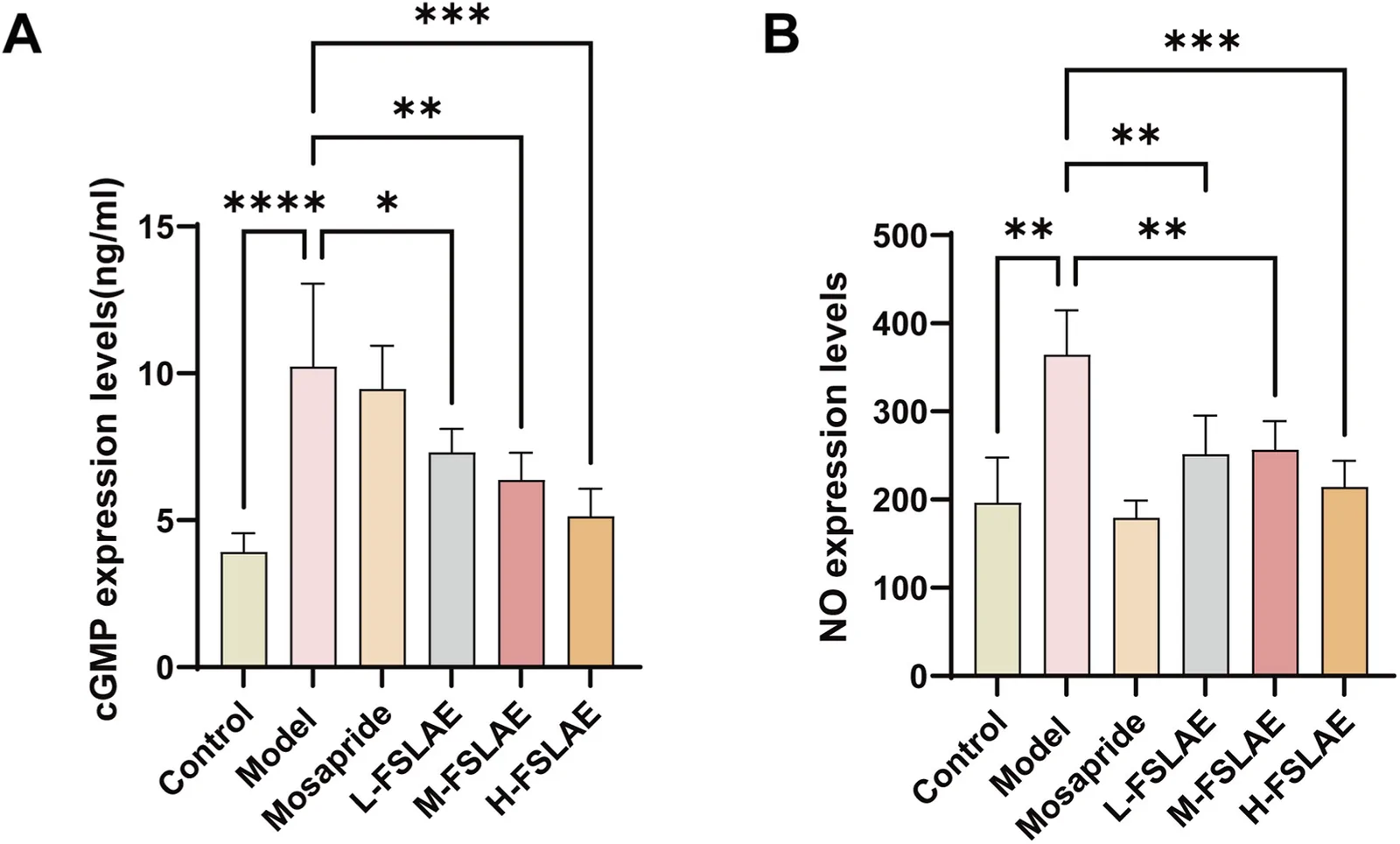
Effects of FSLAE on serum cGMP and NO levels in FD model rats (‾X ± s, n = 10). **(A)** Serum cGMP levels. **(B)** Serum nitric NO levels. *P < 0.05, **P < 0.01, ***P < 0.001, ****P < 0.0001.

Serum cGMP and NO levels in rats were measured by ELISA assay; the results are shown in Figure 9. Relative to the blank control group, the model control group exhibited a highly significant increase in serum cGMP and a highly significant increase in serum NO (both *P* < 0.0001), indicating excessive activation of the NO/cGMP/PKG signaling pathway in FD model rats. Compared with the model control group, all FSLAE doses significantly corrected these abnormalities: cGMP levels declined in a dose-dependent manner (*P* < 0.05, *P* < 0.01, *P* < 0.001), and NO levels also declined in a dose-dependent manner (*P* < 0.01, *P* < 0.001). The high-dose FSLAE group produced the largest reductions in both cGMP and NO.

### 3.9 Effects of FSLAE on the expression of eNOS, p-eNOS, PKG1 and p-PKG1 in gastric tissues of FD model rats

**FIGURE 10:**
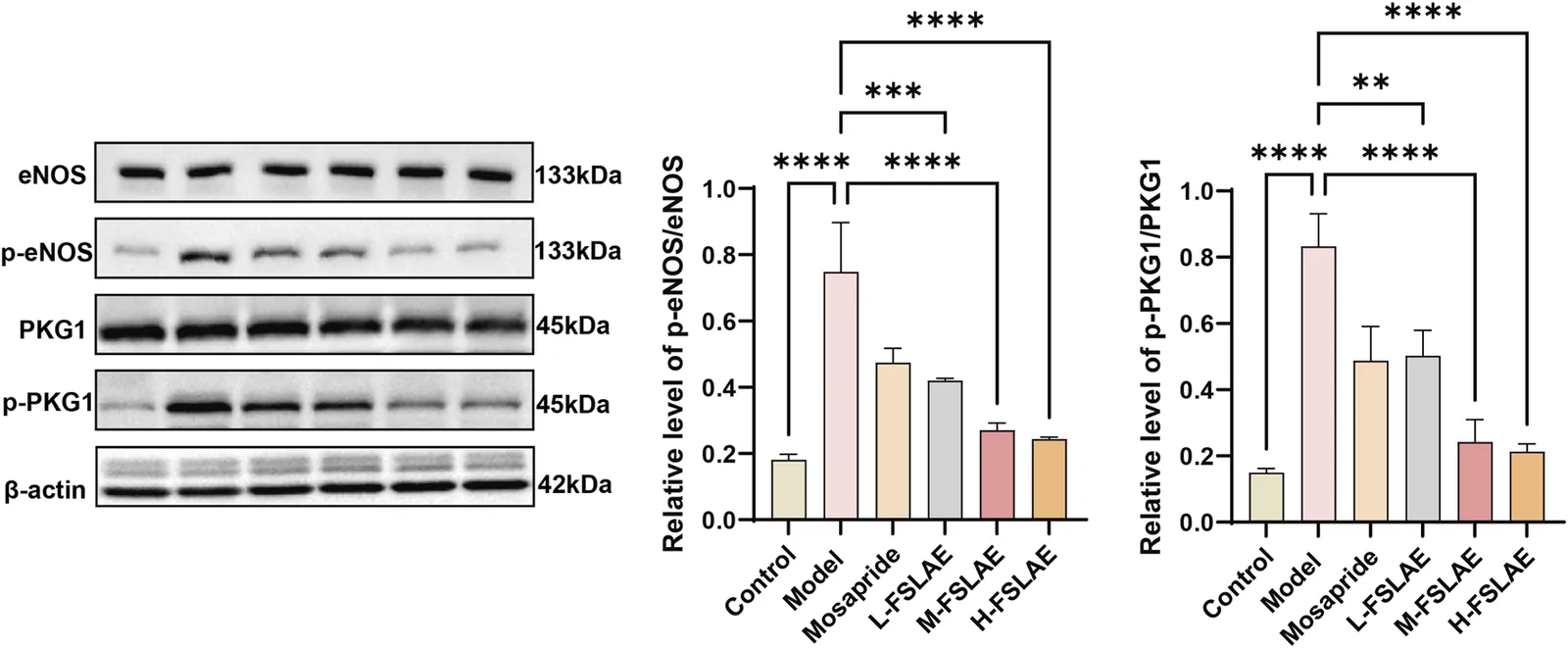
Effects of FSLAE on the protein expression of eNOS/p-eNOS and PKG1/p-PKG1 in gastric tissues of FD model rats (‾X ± s, n = 3). *P < 0.05, **P < 0.01, ***P < 0.001, ****P < 0.0001.

Protein expression of eNOS, p-eNOS, PKG1, and p-PKG1 in rat gastric tissue was measured by Western blot, and the results are presented in Figure 10. Relative to the blank control, the phosphorylation ratios p-eNOS/eNOS and p-PKG1/PKG1 were markedly increased in the model control group (*P* < 0.0001), indicating excessive activation of the NO/cGMP/PKG signaling pathway in gastric tissue of FD model rats. Each dose of FSLAE significantly reduced these phosphorylation ratios compared with the model control in a clear dose-dependent manner (*P* < 0.01, *P* < 0.001, *P* < 0.0001), with higher doses producing greater inhibition of pathway overactivation. These findings suggest that FSLAE may exert therapeutic effects in FD by suppressing excessive activation of the NO/cGMP/PKG signaling pathway.

## 4 Discussion

Leaves of *F*. *sinkiangensis*, a key medicinal organ in the genus Ferula (Apiaceae), contain a complex mixture of natural products that likely underlies their gastroprotective effects. Our prior work used UPLC-Q-TOF-MS to profile blood-absorbed components of FSLAE and identified 23 prototype compounds in plasma. Network pharmacology screening in the present study highlighted five core blood-absorbed ingredients against FD: isoferulic acid (IFA), ferulic acid (FA), 4-methylumbelliferone (4-MU), aesculetin, and scopoletin. FA and IFA are phenolic acids, whereas 4-MU, aesculetin, and scopoletin are coumarins. Both phenolic acids and coumarins have been repeatedly implicated in regulation of gastrointestinal function and thus constitute the principal material basis for FSLAE’s therapeutic effects on FD.

Phenolic acids are an important class of bioactive natural products that modulate gastrointestinal function. FA and IFA, the primary phenolic acid constituents among the blood-absorbed ingredients of FSLAE identified in this study, have pharmacological effects on core pathological features of FD that multiple prior studies have validated. In plants, FA predominantly occurs in an ester-bound form, with only a low proportion in the free state, and it exhibits anti-inflammatory, anti-fibrotic, antioxidant, and vascular endothelial–protective activities (Antonopoulou et al., 2022; Li et al., 2021). BADARY OA et al. reported that FA reversed cisplatin-induced delayed gastric emptying in a dose-dependent manner (Badary et al., 2006). HU X et al. demonstrated that FA restored endothelial nitric oxide synthase (eNOS) expression and synthetic function, increased NO bioavailability, and reduced inflammatory factor levels and p47phox expression in a rat model of metabolic syndrome; additionally, FA mitigated gastrointestinal inflammation by enhancing gut microbial diversity and preserving gastrointestinal microenvironment homeostasis (Hu et al., 2025). XU M et al. demonstrated that FA modulates endothelial cell function and homeostasis, decreases secretion of inflammatory mediators, and attenuates pathological damage in a mouse model of oxidative stress–induced colitis (Xu et al., 2025). As the structural isomer of FA, IFA is another key phenolic acid in FSLAE with established gastric mucosal–protective and intestinal anti-inflammatory activities relevant to FD. LA X et al. reported that IFA promoted synthesis of gastric protective mucus and preserved the physiological function of the gastric mucosal mucus barrier by inhibiting alcohol-induced upregulation of the glycosyltransferase GALNT2 (La et al., 2024). SERRELI G et al. found that IFA suppressed LPS-induced phosphorylation of IκBα, Akt, and p38, reduced inducible nitric oxide synthase (iNOS) expression, and lowered production of NO and cGMP, thereby exerting anti-gastrointestinal inflammatory effects through regulation of the NF-κB signaling pathway (Serreli et al., 2021).

In addition to the core phenolic acids, coumarins comprise another major class of blood-absorbed compounds that mediate the therapeutic effects of FSLAE on FD. The coumarins identified here—4-MU, aesculetin, and scopoletin—are representative molecules with established gastrointestinal regulatory activity. 4-MU displays multiple pharmacological actions, including anti-inflammatory and antioxidant effects and inhibition of hyaluronan (HA) synthesis, and it is widely used clinically to treat biliary spasm in many countries; it also produces indirect anti-inflammatory and anti-cancer effects through HA synthesis inhibition (Fontaine et al., 1968). Prior work showed that 4-MU blocks pathological HA synthesis in a mouse model of intestinal ischemia/reperfusion (I/R), and aberrant HA deposition can disrupt gastrointestinal function via the non-adrenergic non-cholinergic (NANC) pathway by modulating iNOS expression in enteric neurons (Bistoletti et al., 2020). As a coumarin derivative, aesculetin also has pronounced anti-inflammatory activity; it reduces NO, TNF-α, and IL-6 production, downregulates iNOS and NLRP3 expression, and suppresses NF-κB and MAPK pathway activation in LPS-stimulated RAW264.7 cells and in dextran sulfate sodium (DSS)-induced colitis mice (Wang et al., 2022). Scopoletin’s pharmacology has been extensively studied across gastrointestinal diseases, cancer, liver disorders, diabetes, and psychiatric conditions (Gao et al., 2024). Sirima Mahattanadul et al. demonstrated that scopoletin produced a marked therapeutic effect on reflux esophagitis and on both acute and chronic gastric ulcers in rats, with efficacy comparable to lansoprazole and a prokinetic action superior to cisapride, indicating its potential as an active agent for gastroesophageal mucosal injury diseases (Mahattanadul et al., 2011). Wan NurFarahin Wan Osman et al. showed that scopoletin mitigated osteoarthritis through multiple mechanisms, including inhibition of the inflammatory response, suppression of NO production, and reduction of oxidative stress, thereby reinforcing its broad anti-inflammatory pharmacological profile (Wan Osman et al., 2019).

The NO/cGMP/PKG signaling cascade is a key regulator of digestive function and strongly affects both physiological homeostasis and pathological processes in the gastrointestinal tract. Nitric oxide synthase (NOS), the rate-limiting enzyme for NO production, is present in gastrointestinal tissues as three principal isoforms: neuronal NOS (nNOS), endothelial NOS (eNOS), and inducible NOS (iNOS). BARRACHINA M D et al. found that physiologic NO produced by nNOS or eNOS helps maintain gastric mucosal homeostasis by regulating mucosal blood flow, epithelial secretion, and barrier integrity (Barrachina et al., 2001). As a lipophilic gaseous messenger generated by these NOS isoforms, NO diffuses across membranes without a transmembrane receptor and binds soluble guanylate cyclase (sGC) in smooth muscle cells, catalyzing conversion of guanosine triphosphate (GTP) to cGMP. Increased intracellular cGMP binds the regulatory domain of PKG and activates the kinase; activated PKG phosphorylates RhoA and suppresses Rho kinase, thereby enhancing myosin light chain phosphatase (MLCP) activity and reducing Ca^2+^ sensitivity in smooth muscle cells, which produces hypocontractility and impaired gastrointestinal motility. Excessive PKG activation causes sustained phosphorylation of telokin, over-activates MLCP, impairs sustained contraction of gastrointestinal smooth muscle, and decreases gastric emptying and small intestinal transit rates. Elevated circulating NO levels or *in vivo* over-activation of eNOS can also induce duodenal microinflammation and damage the gastric mucosal barrier, which further disrupts gastrointestinal hormone secretion (Yazji et al., 2013; Mori et al., 2022). Network pharmacology and molecular docking analyses predicted that FSLAE may act against FD primarily via the NO/cGMP/PKG signaling pathway, identifying NOS3 and PRKG1 as potential key targets and showing that FSLAE’s systemically absorbed constituents (notably phenolic acids and coumarins) may directly bind to these proteins, thus providing a preliminary molecular rationale for experimental validation. To test this hypothesis, we employed a classic rat model of FD induced by iodoacetamide combined with chronic tail-clamping stress, which reproduces core clinical features of FD, including impaired gastrointestinal motility, low-grade mucosal inflammation, and dysregulated gastrointestinal hormones, and we characterized FSLAE’s therapeutic efficacy in this FD model for the first time. *In vivo*, FSLAE produced dose-dependent improvements in overall condition and weight gain, restored gastric emptying and small intestinal transit, improved gastric mucosal histopathology, rebalanced gastrointestinal hormones, and lowered systemic inflammatory markers. Notably, FSLAE significantly reduced serum IL-4, a cytokine consistently reported as abnormally elevated in FD patients and closely linked to the disease’s low-grade gastrointestinal inflammation (Andersen et al., 2005). ELISA and Western blot analyses showed that FSLAE reversed the abnormal increases in serum NO and cGMP and reduced excessive phosphorylation of eNOS and PKG1 in gastric antral tissue. These findings indicate that FSLAE may relieve FD-like symptoms in rats by inhibiting aberrant activation of the NO/cGMP/PKG signaling pathway and provide preliminary insight into a possible molecular mechanism for its anti-FD effects.

Most prior research on *F*. *sinkiangensis* has concentrated on its oleoresin and roots, which are rich in volatile oils, while the medicinal potential of its leaves has been largely overlooked. This study is the first to systematically define the pharmacodynamic material basis of FSLAE against FD and to demonstrate its multi-component, multi-target, and multi-pathway regulatory characteristics using network pharmacology, thereby filling a knowledge gap regarding this plant part in the study of functional gastrointestinal disorders. In addition, the study furnishes rigorous scientific support for the traditional clinical use of *F*. *sinkiangensis* leaves in treating gastrointestinal complaints and identifies novel candidate active compounds and potential intervention targets for developing effective, low-toxicity anti-FD therapeutics, addressing the clinical limitations of current first-line prokinetic agents (e.g., domperidone) that can carry cardiovascular risks.

This study has several limitations that warrant further investigation. First, we performed detailed validation only for the NO/cGMP/PKG signaling pathway; potential synergistic contributions of other FD-related pathways identified by network pharmacology, including PI3K-Akt and MAPK, to FSLAE-mediated anti-FD effects remain unexamined. Second, although molecular docking predicted binding of core blood-absorbed ingredients to target proteins, the direct pharmacodynamic effects and precise molecular mechanisms of these individual compounds against FD require systematic *in vivo* and *in vitro* validation. Third, FD is a complex, multifactorial disorder whose pathophysiology extends well beyond impaired gastrointestinal motility, which this study primarily addressed. Although FSLAE improved gastric emptying and small intestinal transit in the iodoacetamide plus chronic tail-clamping stress model, our design did not examine several other central pathophysiological features of FD. We did not assess visceral hypersensitivity, a hallmark of FD that strongly influences symptom severity. We also did not evaluate gastric accommodation dysfunction, which is closely linked to early satiation and postprandial fullness. Our analysis was restricted to peripheral tissues and serum markers, so we did not investigate FSLAE effects on vagal signaling or on central nervous system processing of visceral sensations. We furthermore did not assess anxiety and depression comorbidities that often exacerbate FD symptoms. Nor did we examine whether gut microbiota dysbiosis—a key mediator of gastrointestinal motility and brain–gut axis function—contributes to the therapeutic effects of FSLAE, despite assessing low-grade gastrointestinal inflammation.

Future work will address these limitations by systematically evaluating the pharmacodynamics of FSLAE’s core monomeric constituents and their synergistic interactions. We will dissect crosstalk between the NO/cGMP/PKG pathway and other identified signaling cascades. We will comprehensively assess FSLAE’s effects on additional core FD pathophysiological features, including visceral hypersensitivity, gastric accommodation, vagal nerve function, and affective comorbidities. We will also investigate the role of the gut microbiota and the microbiota–gut–brain axis in mediating FSLAE’s therapeutic effects. Together, these studies will yield a more complete understanding of FSLAE’s holistic anti-FD mechanisms and will strengthen the rationale for clinical translation.

## Data Availability

The original contributions presented in the study are included in the article/Supplementary Material, further inquiries can be directed to the corresponding authors.
