# Supplementary material for: Investigation of the putative mechanism by which Ferula sinkiangensis leaves alleviate functional Dyspepsia–like symptoms in rats via modulation of the NO/cGMP/PKG signaling pathway
Source: Front Pharmacol. 2026 Sep 1;17:1843817. doi: 10.3389/fphar.2026.1843817 (PMC13574696; doi:10.3389/fphar.2026.1843817)
Supplement: Supplementary file 1 [file DataSheet1.pdf]

1 Methods

1.1 Preparation of Ferula sinkiangensis Leaf Aqueous Extract (FSLAE)

A 200 g batch of processed medicinal slices of F. sinkiangensis leaves was crushed and passed through a 40-mesh sieve. Ultra-pure water equal to 10 times the mass of the plant material was added to the powder, which was soaked for 1 h. Extraction was carried out by heating in an 80 °C water bath for 1.5 h, followed by hot filtration. The extraction was repeated three times, and the filtrates from all rounds were combined. The combined filtrate was concentrated under reduced pressure in a 65 °C water bath to yield a fluid extract with a crude drug concentration of 2 g mL^{-1}. The extract was sealed in ampoules, stored at -20 °C, and used for compositional analysis and animal experiments.

1.2 Preparation of Test Solution

A 100 µL aliquot of F. sinkiangensis leaf fluid extract was combined with 900 µL of 50% methanol in water, vortexed for 30 s, and held at 4 °C for 2 h. The mixture was centrifuged at 15,000 rpm and 4 °C for 15 min, and the supernatant was filtered through a 0.22 µm organic-phase microporous membrane. The filtrate was transferred to a sample vial for subsequent analysis.

1.3 Preparation of Plasma Samples

Rats were acclimatized for 7 days before the experiment and then randomly assigned to a blank control group (n=10) and an administration group (n=10). Rats in the administration group received F. sinkiangensis leaf aqueous extract by intragastric administration at a dose of 10 g kg^{-1} body weight, while rats in the blank control group received an equal volume of normal saline by intragastric administration. Dosing occurred twice daily (morning and evening) for 2 consecutive days. On day 3, blood samples were collected from the retro-orbital venous plexus at 0.5, 1, 2, and 4 h after the final intragastric administration and were immediately transferred into anticoagulant EP tubes containing EDTA-K_2. Samples stood at 4 °C for 2 h and were then centrifuged at 3500 rpm (4 °C) for 15 min to separate the upper plasma layer. Plasma aliquots were stored at -80 °C until further processing.

1.4 Plasma Sample Processing

Plasma samples collected at different time points from each group were pooled separately. A 300 μL aliquot of the pooled plasma was mixed with three volumes of pre-cooled methanol, vortexed for 3 min, sonicated in an ice bath for 20 min, and allowed to stand at 4 °C for 10 min. The mixture was centrifuged at 15,000 rpm (4 °C) for 15 min, and the supernatant was transferred to a centrifugal concentration tube. The solvent was evaporated to dryness under vacuum at 45 °C. The residue was reconstituted in 200 μL of 50% methanol, vortexed for 3 min, and centrifuged at 15,000 rpm (4 °C) for 15 min. The supernatant was filtered through a 0.22 μm microporous membrane and transferred to a sample vial for analysis.

1.5 Chromatographic and Mass Spectrometric Conditions

Chromatographic separation was performed on an ACQUITY UPLC® HSS T3 column (2.1 mm × 100 mm, 1.8 μm; Waters, Milford, MA, USA). The mobile phase comprised 0.1% formic acid in water (phase A) and acetonitrile with 0.1% formic acid (phase B). The gradient program was: 0–1 min, 0% B; 1–2 min, linear to 30% B; 2–12 min, linear to 50% B; 12–21 min, linear to 100% B; 21–26 min, hold at 100% B; 26–26.1 min, linear to 0% B; 26.1–30 min, hold at 0% B for column equilibration. The flow rate was 0.3 mL min^{-1}, column temperature was 40 °C, injection volume was 6 μL, and the sample chamber was maintained at 4 °C.

Mass spectrometric data were acquired in both positive and negative ionization modes using an electrospray ionization (ESI) source. Key parameters were: ion source temperature, 500 °C; Ion Source Gas 1 (GS1), 60 psi; Ion Source Gas 2 (GS2), 60 psi; curtain gas (CUR), 45 psi; capillary voltage, +5500 V (positive mode) and −4500 V (negative mode); declustering potential (DP), 60 V; collision energy (CE) for TOF MS scan, 10 V; CE for product ion scan, 40 V; collision energy spread (CES), 20 V. The mass scan range was m/z 90–1500 for TOF MS and m/z 50–1500 for product ion scans, with a total acquisition time of 30 min. Information-dependent acquisition (IDA) triggered product ion scans for the top 18 singly charged ions with intensity >100 counts per second (cps) in the TOF MS survey scan, and dynamic background subtraction (DBS) was enabled. Data were acquired with Analyst TF 1.7.1 software (AB Sciex, Framingham, MA, USA).

1.6 Identification of Chemical Constituents and Blood-Absorbed Components

Raw mass spectrometric data were processed with MSDIAL for peak alignment, retention time correction, and peak area extraction, while background noise was removed based on chromatographic peak signal-to-noise ratio (S/N). Preprocessed data from the aqueous extract, blank plasma, and drug-containing plasma, acquired in both positive and negative ionization modes, were imported into UNIFI for matching against the Traditional Chinese Medicine Database BPTCM 2.0. Structural assignments were made by matching MS^2 fragment ions to entries in PubChem, HMDB, and the published literature. Matching parameters were: precursor ion (MS^1) mass error < 0.02 Da, product ion (MS^2) mass error < 0.05 Da, and total matching score > 70. Identification reliability was evaluated by the MS^2 fragment matching Score (maximum = 1); Score > 0.7 was considered credible. By comparing base peak chromatograms (BPCs) of the aqueous extract, blank plasma, and drug-containing plasma, endogenous components and false positives from blank plasma were excluded. Compounds found only in drug-containing plasma were designated as the blood-absorbed components of FSLAE.

2 Results

2.1 Identification of Blood-Absorbed Prototype Components of FSLAE

Using the method in Section 1.6, we characterized 191 chemical constituents in FSLAE. From those, 23 prototype components absorbed into blood were identified by comparing drug-containing rat plasma, the in vitro chemical profile of FSLAE, and blank rat plasma. These absorbed components primarily comprised phenolic acids, coumarins, fatty acids, sesquiterpenes, and flavonoids. Detailed information on the identified components appears in Table 1, and the total ion chromatograms (TICs) of FSLAE and the corresponding drug-containing plasma samples are shown in Fig.1.

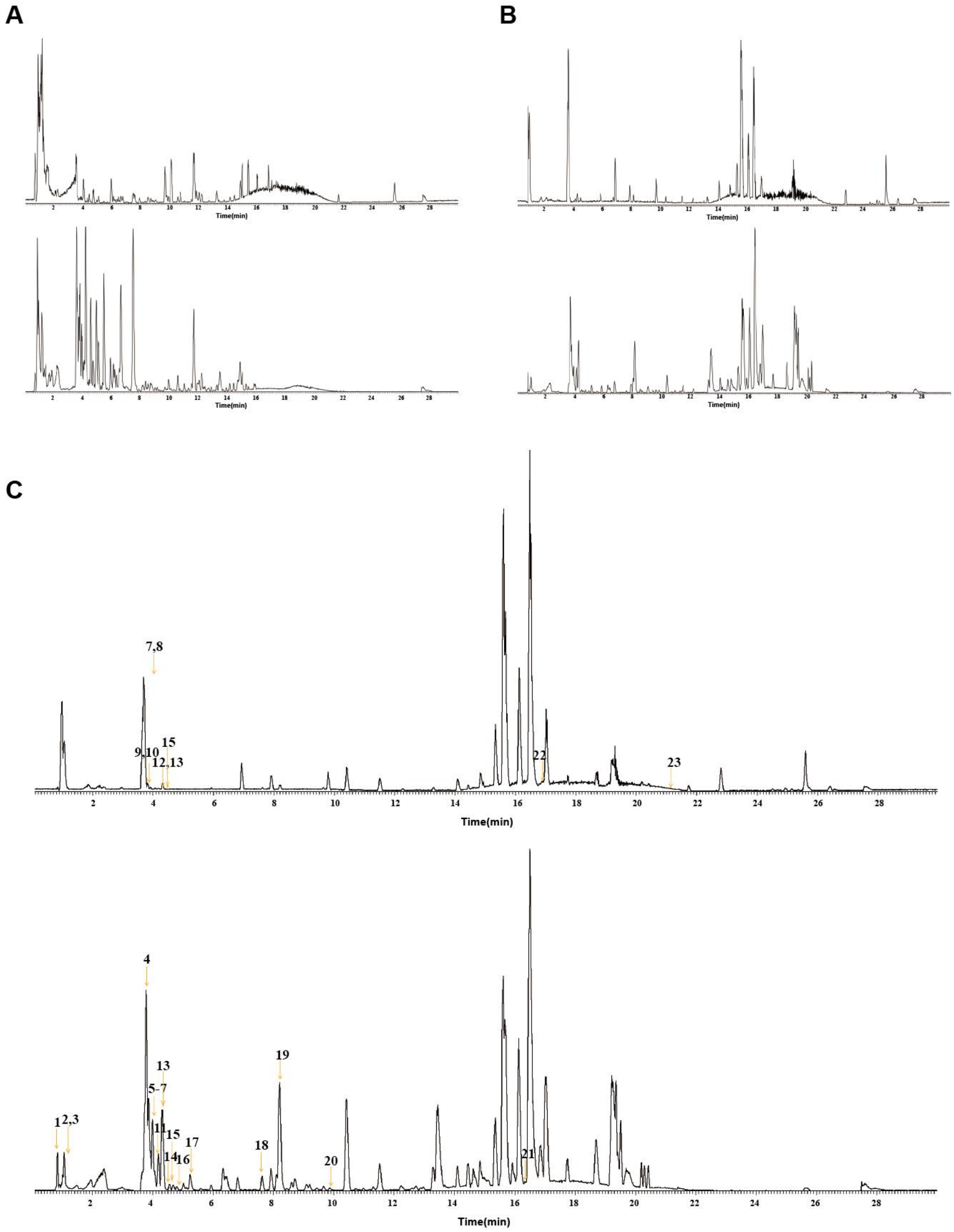

Note: A. FSLAE extract in vitro; B. rat blank serum; C. rat serum after administration. (Upper panel: positive ion mode; lower panel: negative ion mode).

Fig 1 Total Ion Chromatogram (TIC) Profiles of FSLAE and FSLAE-Administered Plasma

Table 1 Analysis of Blood-borne Components of FSLAE

No.	t_R/min	Compound	Molecular Formula	Ion Mode	Theoretical m/z	Mass Error (ppm)	MS² Fragments
1	0.981	D-Sorbitol	$C_6H_{14}O_6$	$[M-H]^-$	181.071 7	3.203 2	55.018 0, 58.006 3, 59.013 8, 71.013 9, 73.028 1, 85.030 8, 89.024 4, 101.024 2

2	1.191	5,6,2',3',6'-Pentamethoxyflavone	$C_{20}H_{20}O_7$	$[M-H]^-$	371.113 7	-6.817 3	55.019 2, 58.006 4, 59.013 9, 71.014 1, 85.028 4, 87.009 8, 119.034 2, 371.118 8
3	1.223	Turanose	$C_{12}H_{22}O_{11}$	$[M-H]^-$	341.108 9	3.869 7	55.019 3, 57.034 1, 58.006 5, 59.014 0, 71.014 1, 85.028 5, 89.024 7, 119.035 8
4	3.75	2-Isopropylmalic acid	$C_7H_{12}O_5$	$[M-H]^-$	175.060 9	4.169 9	57.035 1, 59.015 0, 69.071 2, 83.049 3, 85.065 9, 87.045 1, 113.061 2, 115.041 6
5	3.828	3-p-Coumaroylquinic acid	$C_{16}H_{18}O_8$	$[M-H]^-$	337.093 5	5.992 4	57.036 1, 59.013 9, 67.018 4, 71.014 0, 85.029 6, 87.008 3, 93.035 3, 191.056 4
6	3.83	7-Hydroxycoumarin sulfate ester	$C_9H_6O_6S$	$[M-H]^-$	240.981 0	3.734 7	65.002 9, 77.040 6, 89.039 6, 93.035 9, 105.034 3, 117.033 7, 121.027 9, 133.030 7
7	3.857	3-O-Feruloylquinic acid	$C_{17}H_{20}O_9$	$[M-H]^-$|$[M+H]^+$	367.103 3|369.118 2	-0.844 5	57.035 1, 58.006 4, 59.013 9, 67.019 7, 87.008 5, 93.035 4, 134.038 5, 173.047 6
8	3.874	Isoferulic acid	$C_{10}H_{10}O_4$	$[M+H-H2O]^+$	177.054 0	-5.083 2	77.037 8, 78.045 6, 89.039 1, 91.054 1, 106.043 3, 117.032 6, 134.032 5, 145.029 8
9	3.9	Aesculetin	$C_9H_6O_4$	$[M-H]^-$|$[M+H]^+$	177.019 3|179.033 9	-5.762 1	77.039 6, 89.038 5, 105.034 5, 107.014 7, 121.028 1, 123.045 2, 133.029 3, 149.026 6
10	3.929	Geraniol	$C_{10}H_{18}O$	$[M+Na]^+$	177.125 0	-4.177 8	89.039 2, 117.035 8
11	4.006	Anisic acid	$C_8H_8O_3$	$[M-H]^-$	151.040 0	8.275 9	92.019 6
12	4.45	4-Methylumbelliferone	$C_{10}H_8O_3$	$[M+H]^+$	177.054 5	-12.538 5	63.022 1, 77.039 0, 89.039 0, 91.054 1, 103.053 1, 106.041 8, 117.034 1, 134.037 3
13	4.455	Ferulic acid	$C_{10}H_{10}O_4$	$[M-H]^-$|$[M+H]^+$	193.050 6|195.065 2	6.164 2	78.047 9, 89.037 6, 91.059 3, 106.044 5, 117.033 9, 133.031 9, 134.037 1, 145.029 4
14	4.46	Luteolin 4'-glucoside	$C_{21}H_{20}O_{11}$	$[M-H]^-$	447.093 3	9.215 1	285.039 3, 447.093 9
15	4.563	Scopoletin	$C_{10}H_8O_4$	$[M+H]^+$|$[M-H]^-$	193.049 3|191.035 0	-1.087 8	53.040 2, 66.046 1, 77.039 0, 81.033 8, 89.037 7, 122.036 0, 133.027 6, 193.049 9
16	4.736	Cinnamic acid	$C_9H_8O_2$	$[M-H]^-$	147.045 1	4.284 4	77.037 7, 103.053 6, 119.054 2
17	5.105	2-methylbutyl (acetyloxy)acetate	$C_9H_{16}O_4$	$[M-H]^-$	187.097 7	14.110 3	59.014 6, 99.081 8, 127.077 1
18	7.73	Traumatic Acid	$C_{12}H_{20}O_4$	$[M-H]^-$	227.129 0	7.132 5	165.131 4, 183.130 6, 183.132 5, 183.136 3, 183.138 2, 183.140 1, 183.142 0, 183.143 9
19	8.446	3,16-dihydroxypalmitic acid	$C_{16}H_{32}O_4$	$[M-H]^-$	287.223 3	3.725 3	99.081 1, 113.095 5, 127.112 8, 129.092 6, 141.129 4, 223.204 7, 269.213 8, 287.223 0
20	10.151	4-Methoxy-5-nonylbenzene-1,3-diol	$C_{16}H_{26}O_3$	$[M-H]^-$	265.181 0	-6.448 4	265.163 7
21	11.785	Pulchelloid D	$C_{25}H_{34}O_8$	$[M+H]^+$|$[M-H]^-$	461.218 3|461.218 3	0.715 5	161.023 4, 161.025 2, 161.028 8, 161.034 1

22	16.927	Citronellyl acetate	$C_{12}H_{22}O_2$	$[M+H]^+$	199.169 2	-1.154 8	55.055 4, 69.071 4
23	21.183	α-iso-cubebenol	$C_{15}H_{24}O$	$[M-H]^-$	219.175 5	8.714 5	205.122 8
