# Supplementary material for: Investigation of the putative mechanism by which Ferula sinkiangensis leaves alleviate functional Dyspepsia–like symptoms in rats via modulation of the NO/cGMP/PKG signaling pathway
Source: Front Pharmacol. 2026 Sep 1;17:1843817. doi: 10.3389/fphar.2026.1843817 (PMC13574696; doi:10.3389/fphar.2026.1843817)

eNOS

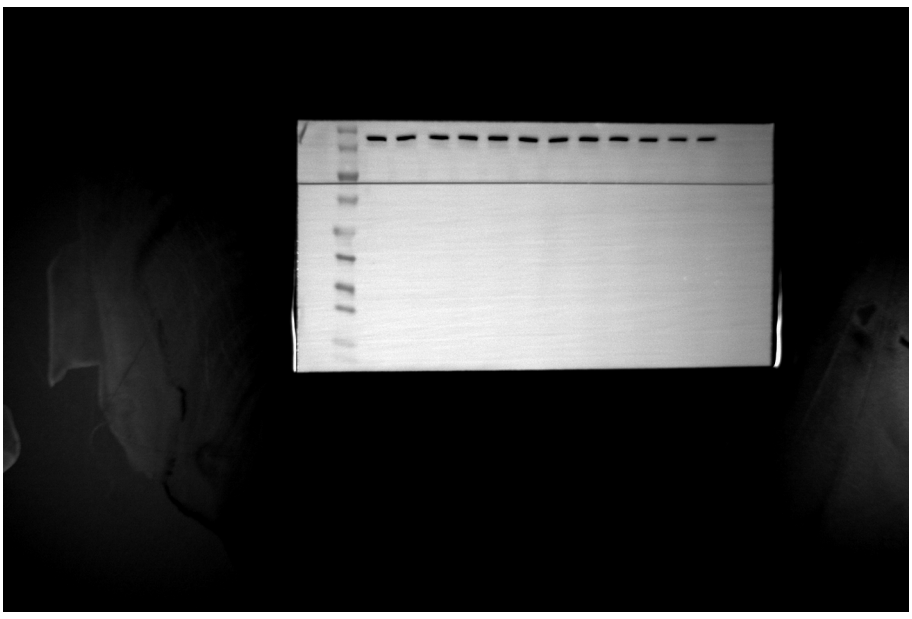

p-eNOS

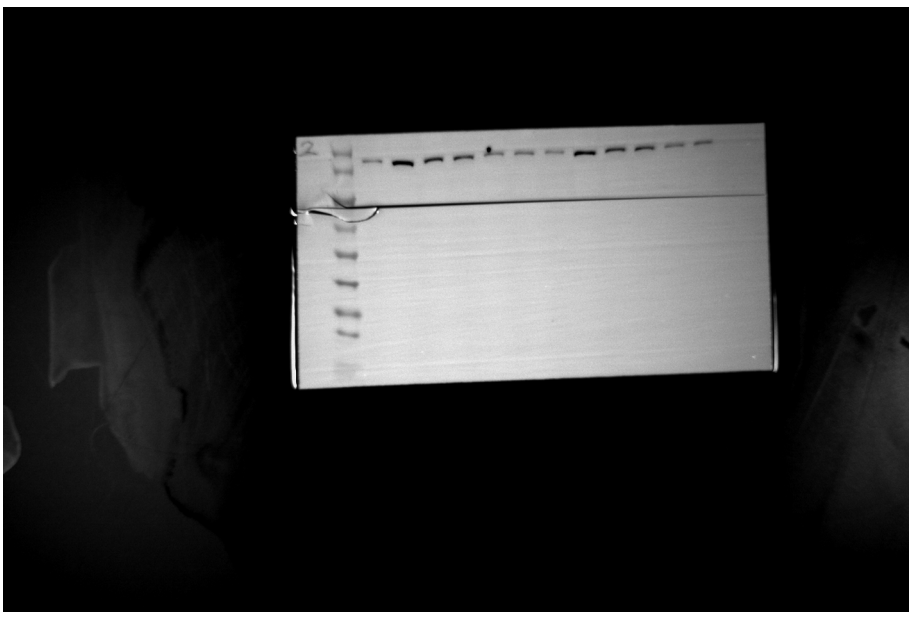

PKG1

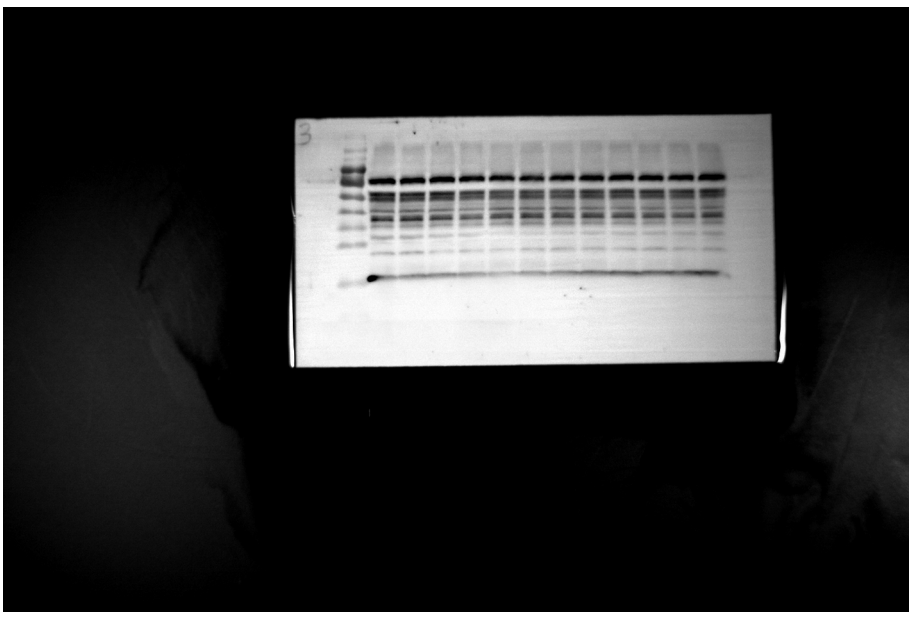

p-PKG1

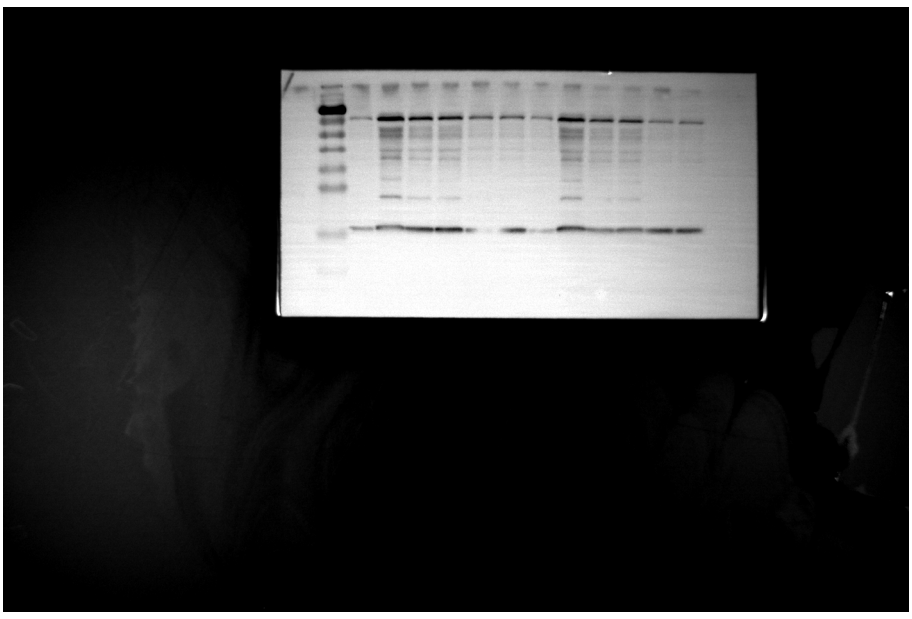

β -actin

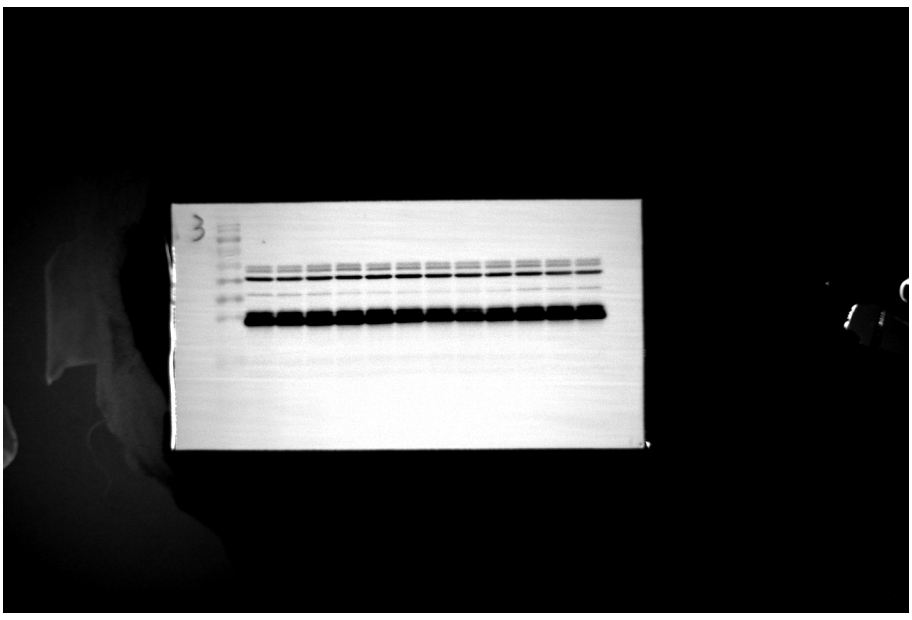

Supplement: Supplementary file 2 [file DataSheet2.pdf]
